# The DDX39B/FUT3/TGFβR-I axis promotes tumor metastasis and EMT in colorectal cancer

**DOI:** 10.1038/s41419-020-03360-6

**Published:** 2021-01-12

**Authors:** Chengcheng He, Aimin Li, Qiuhua Lai, Jian Ding, Qun Yan, Side Liu, Qingyuan Li

**Affiliations:** Guangdong Provincial Key Laboratory of Gastroenterology, Department of Gastroenterology, Nanfang Hospital, Southern Medical University, Guangzhou, Guangdong 510515 China

**Keywords:** Colorectal cancer, Mechanisms of disease

## Abstract

DDX39B is a member of the DEAD box (DDX) RNA helicase family required for nearly all cellular RNA metabolic processes. The exact role and potential molecular mechanism of DDX39B in the progression of human colorectal cancer (CRC) remain to be investigated. In the present study, we demonstrate that DDX39B expression is higher in CRC tissues than in adjacent normal tissues. Gain- and loss-of-function assays revealed that DDX39B facilitates CRC metastasis in vivo and in vitro. Mechanistically, RNA-sequencing (RNA-seq) and RNA-binding protein immunoprecipitation-sequencing (RIP-seq) showed that DDX39B binds directly to the FUT3 pre-mRNA and upregulates FUT3 expression. Splicing experiments in vitro using a Minigene assay confirmed that DDX39B promotes FUT3 pre-mRNA splicing. A nuclear and cytoplasmic RNA separation assay indicates that DDX39B enhances the mRNA export of FUT3. Upregulation of FUT3 accelerates the fucosylation of TGFβR-I, which activates the TGFβ signaling pathway and eventually drives the epithelial–mesenchymal transition (EMT) program and contributes to CRC progression. These findings not only provide new insight into the role of DDX39B in mRNA splicing and export as well as in tumorigenesis, but also shed light on the effects of aberrant fucosylation on CRC progression.

## Introduction

Colorectal cancer (CRC) is among the most common malignant cancers worldwide, and the high mortality rate of CRC has made it a major health burden^1^. Although a certain degree of progress has been made in the diagnosis and treatment of CRC, the overall prognosis of patients with CRC remains low, as tumor relapse and metastasis pose a great challenge to both clinicians and patients. Thus, considerable research on the molecular understanding of CRC metastasis is urgently needed. The TGFβ signaling pathway plays a crucial role in cancer metastasis via angiogenesis, the epithelial–mesenchymal transition (EMT) program, and extracellular matrix (ECM) degradation^2–4^. Therefore, the TGFβ signaling pathway may render certain genes capable of promoting cancer metastasis^5–7^.

Members of the DEAD box (DDX) RNA helicase family are characterized by the presence of a conserved DEAD motif (Asp-Glu-Ala-Asp) and required for virtually all cellular RNA metabolic processes, including transcription, splicing, ribosome biogenesis, nuclear export, translation, and degradation^8,9^. Hence, the deregulation of DDXs may result in the disruption of RNA processing and may exert detrimental effects on the expression of certain key genes, such as oncogenes and tumor suppressors^10–12^. Therefore, cancer cells may rely on DDXs to attain increased expression of oncogenes and decreased production of tumor suppressors to promote cancer survival.

DDX39B is a well-studied RNA helicase in terms of its enzymatic activity and properties in RNA metabolism^13,14^. Notably, DDX39B is a pivotal splicing factor that promotes recruitment of the spliceosome and RNA export adaptor complexes, such as the exon–junction complex (EJC) and transcription–export (TREX) complex^15–17^. These complexes are recruited to mRNA in the nucleus and assist the cytoplasmic localization of RNA. However, the function of DDX39B in diseases such as cancer is largely unexplored and remains to be studied. DDX39A, a paralog of DDX39B, has been shown to have cancer-promoting activity in lung squamous-cell carcinoma, urinary bladder cancer, human malignant pleural mesothelioma, and hepatocellular carcinoma^18–22^. These findings prompted us to investigate whether DDX39B has oncogenic or tumor-suppressive potential in the progression of CRC.

In the current study, we explored the exact role and molecular interactions of DDX39B in the development of CRC. Herein, we report that DDX39B promotes CRC metastasis in vitro and in vivo. Moreover, we found that DDX39B modulates FUT3 expression by regulating the mRNA splicing and nuclear export of FUT3, which results in the aberrant fucosylation of TGFβR-I and activates the TGFβ signaling pathway. Taken together, our results demonstrate a mechanism for the involvement of the DDX39B/FUT3/TGFβR-I axis in the progression of CRC.

## Results

### DDX39B is dysregulated in CRC tissues and cells

**Fig. 1:**
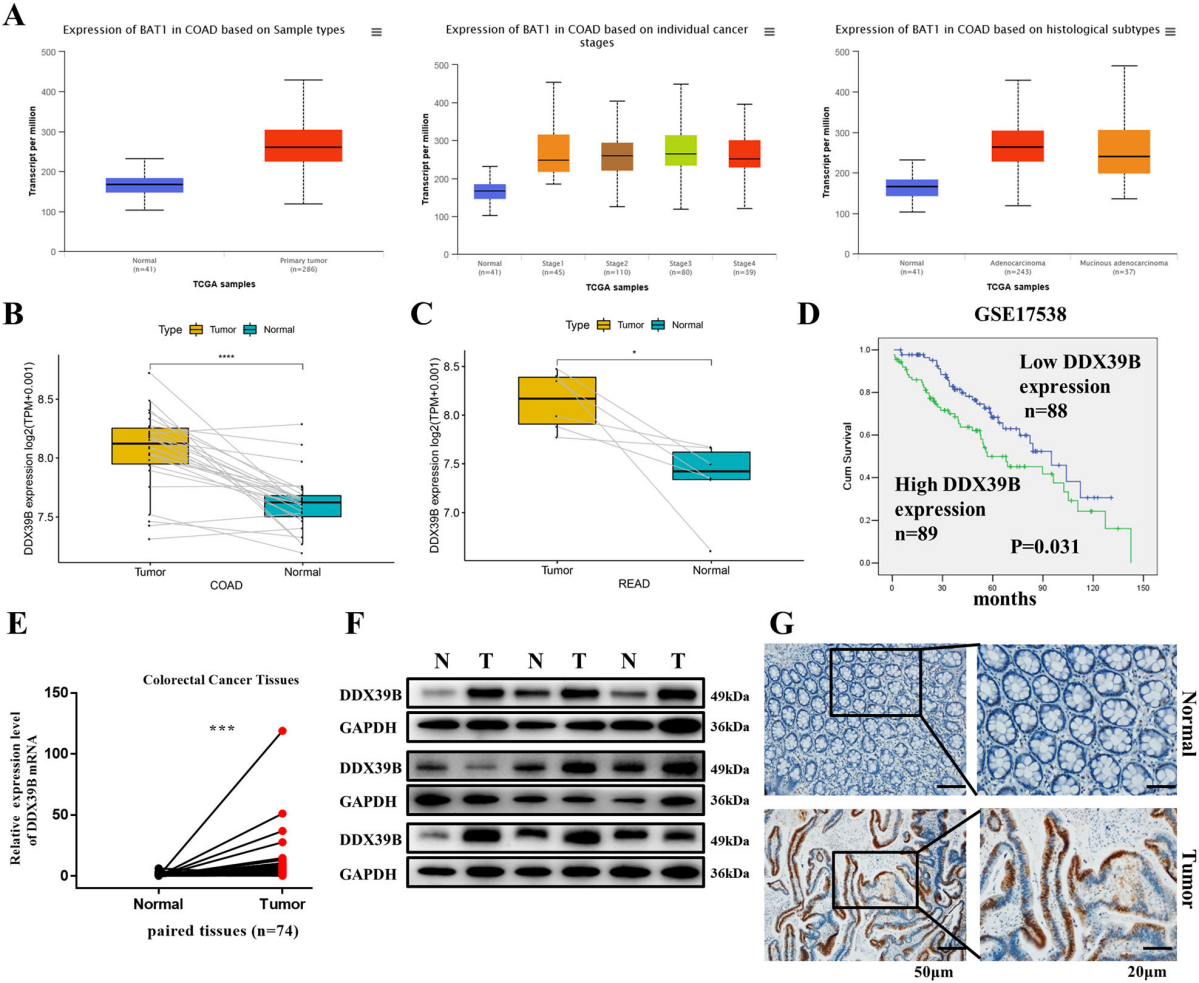
DDX39B expression in colorectal cancer and normal tissues. **A** DDX39B/BAT1 expression in COAD (different histological types and cancer stages from the TCGA Dataset) and normal tissues (unpaired). **B**, **C** DDX39B expression in paired COAD/READ and normal tissues (TCGA Database, *n* = 26/*n* = 6). **D** Overall survival with low/high DDX39B expression, obtained from a colorectal cancer set of GSE17538. **E** DDX39B mRNA expression in colorectal cancer tissues and paired non-cancerous tissues (*n* = 74). **F** DDX39B protein expression in nine paired colorectal cancer and normal tissues. **G** IHC analysis of paired colorectal cancer and adjacent non-tumor tissues; scale bars, 20/50 μm. COAD colon adenocarcinoma, READ rectum adenocarcinoma.

Analysis of data from The Cancer Genome Atlas (TCGA) database^23^ suggested that DDX39B (also called BAT1) was overexpressed in CRC samples compared with normal samples (paired and unpaired tissues) and that DDX39B expression was higher to varying degrees in cancers of different histological types and stages (Fig. 1A-C). Moreover, a Kaplan–Meier analysis of the overall survival of CRC patients revealed that patients with high DDX39B expression levels had a shorter overall survival time than patients with low DDX39B expression levels (Fig. 1D, *P* = 0.031). A similar result was obtained from the data in the PROGgeneV2-Pan Cancer Prognostic Database (Fig. S1B, *P* = 0.014).

To evaluate DDX39B expression levels in CRC, we performed western blotting, qRT-PCR, and IHC. DDX39B mRNA in 74 paired CRC and normal tissues were detected by qPCR, and it was shown that CRC tissues exhibited upregulated DDX39B compared with adjacent normal tissues (Fig. 1E). The detection of nine pairs of human CRC and normal tissues by western blotting confirmed this finding (Fig. 1F). By means of IHC, we also observed increased expression of DDX39B in CRC tissues compared with matched normal tissues (Fig. 1G). Furthermore, we detected the expression level of DDX39B in six CRC cell lines by western blotting. DDX39B expression was highest in RKO cells, low in HT29, HCT116, SW620, and LoVo cells, and moderate in SW480 cells (Fig. S1A).

### DDX39B enhances the migration and invasion of CRC cells in vitro and in vivo

**Fig. 2:**
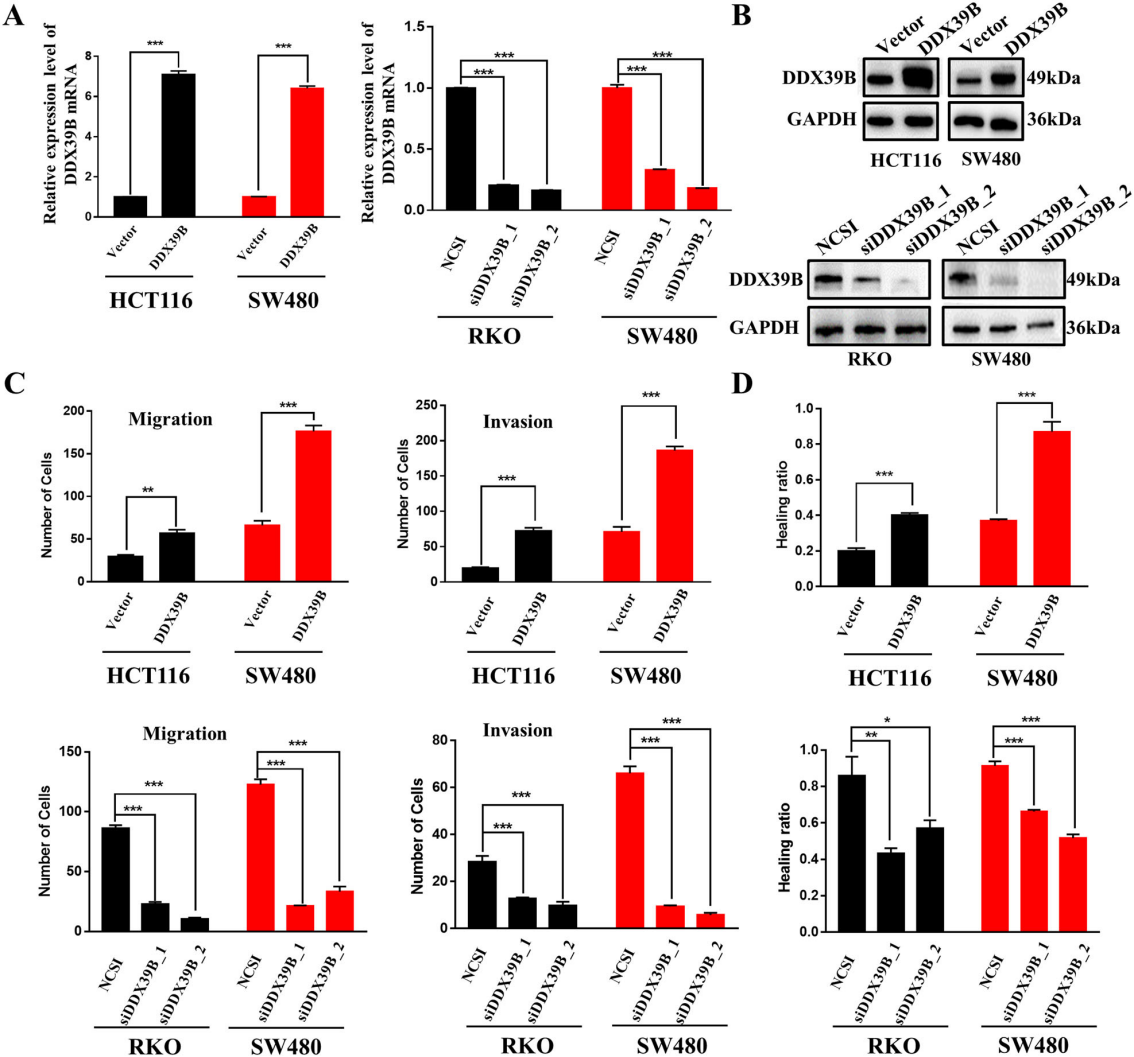
DDX39B enhances the migration and invasion of CRC cells in vitro. **A**, **B** Transfected efficiency was determined using qPCR and western blotting in HCT116, SW480, and RKO cells with overexpressed or silenced DDX39B; Error bars, SD. **C** Migration and invasion abilities in HCT116, SW480, and RKO cells with DDX39B overexpression or silencing (Transwell assay); error bars, SD. **D** Migration capacity in HCT116, SW480, and RKO cells with DDX39B overexpression or silencing (wound healing assay); error bars, SD.

To explore the biological function of DDX39B in CRC, we first examined the transfection efficiency in CRC cells with DDX39B overexpression and knockdown using western blotting, qRT-PCR (Fig. 2A, B), and immunofluorescence (IF) (Fig. S1C). To detect the migration and invasion of CRC cells with DDX39B overexpression or silencing, Transwell and wound healing assays were performed. As shown in Fig. 2C, D and Fig. S1D–F, the numbers of migrated and invaded cells were higher in HCT116/DDX39B and SW480/DDX39B cells than in HCT116/Vector and SW480/Vector cells. Upon depletion of DDX39B, we observed a significant decrease in cell migration and invasion in the RKO/siDDX39B_1, RKO/siDDX39B_2, SW480/siDDX39B_1, and SW480/siDDX39B_2 groups. Taken together, DDX39B enhances the migration and invasion capacities of CRC cells.

**Fig. 3:**
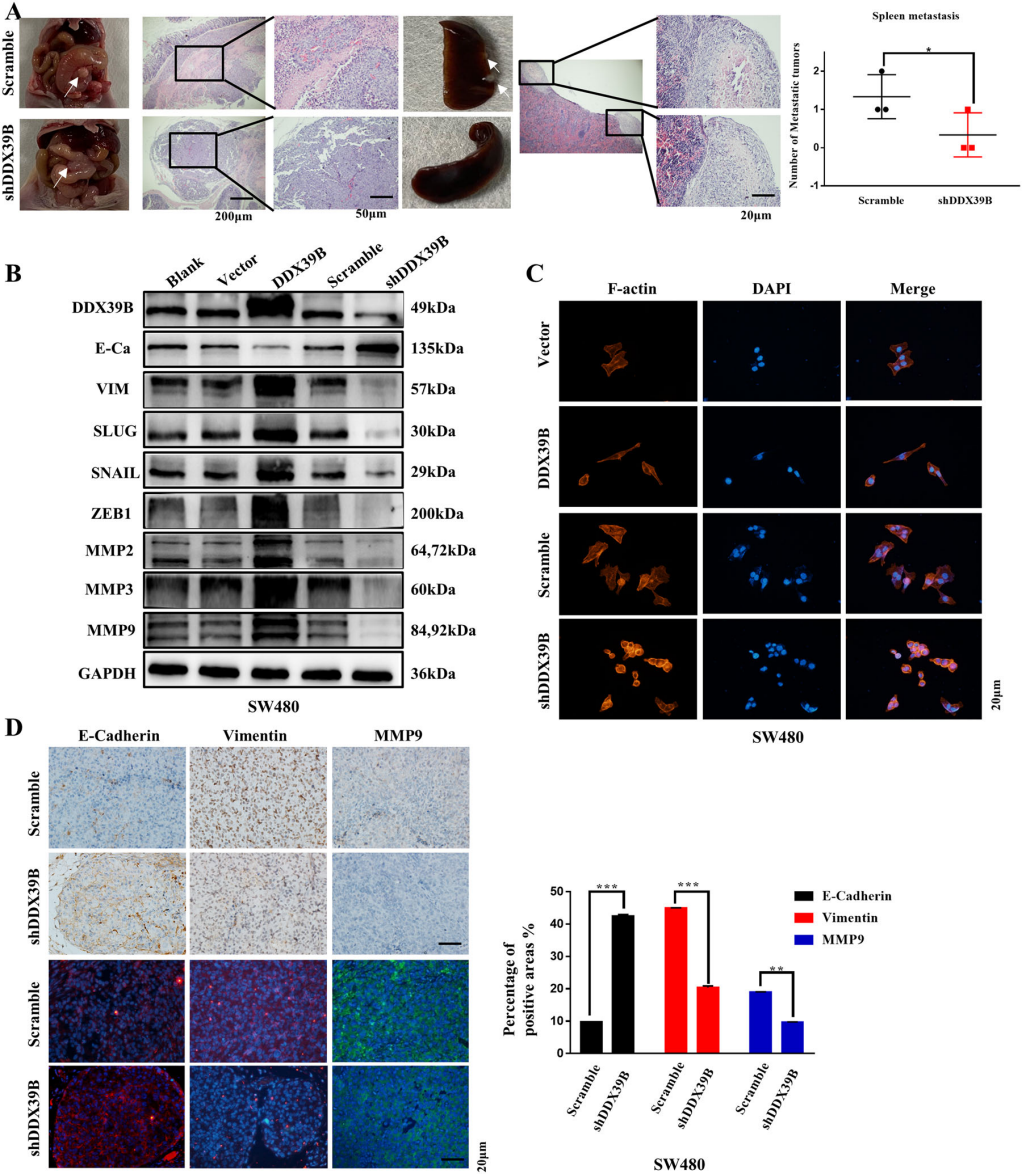
DDX39B promotes CRC metastasis in vivo and EMT. **A** HE staining of CRC orthotopic transplantation tumor and spleen metastasis in SW480/Scramble and SW480/shDDX39B groups. Spleen metastatic numbers were compared between these groups; error bars, SD; scale bars, 20/50/200 μm. **B** EMT marker genes and MMP expression in SW480 cells with DDX39B overexpression and knockdown. **C** Cytoskeleton in SW480 cells with DDX39B overexpression and knockdown, assessed by FITC-phalloidin staining; scale bars, 20 μm. **D** IHC and IF of orthotopic transplantation tumor in mice. E-cadherin, Vimentin, and MMP9 expression levels were compared between SW480/Scramble and SW480/shDDX39B groups; scale bars, 20 μm. Relevant pathological scores of each were also obtained; error bars, SD.

To validate our findings in vivo, an orthotopic transplantation model of CRC was established in nude mice. Notably, more spleen metastases were observed in the SW480/Scramble group compared with the SW480/shDDX39B group (Fig. 3A).

### DDX39B facilitates the EMT program in CRC

Since the EMT program is a key process that contributes to CRC metastasis^24^, we detected the role of EMT in CRC cells with DDX39B overexpression and silencing. As the EMT program may be impaired in microsatellite unstable cells, such as in the HCT116 cell line^25^, we conducted the EMT assay in SW480 cells. Western blotting showed the increased expression levels of Vimentin, MMP2/3/9, SNAIL, SLUG, and ZEB1 and decreased expression level of E-cadherin in the overexpressed DDX39B group compared to the control group. In contrast, the opposite effect was observed in the silenced DDX39B group (Fig. 3B). Additionally, qRT-PCR was used to detect the mRNA expression levels of EMT marker genes and MMPs, the results of which were similar (Fig. S2A, B). Furthermore, F-actin staining was performed to evaluate the transformation of the cytoskeleton. SW480/DDX39B cells exhibited a spindle-like, fibroblastic morphology with the rearrangement of F-actin fibers, while cells in SW480/shDDX39 group exhibited a round or flat morphology (Fig. 3C).

Consistent with these findings, IHC and IF analysis of orthotopic cecal tumors from the mice revealed the increased expression of Vimentin and MMP9 and decreased expression of E-cadherin in the SW480/Scramble group compared with the SW480/shDDX39B group (Fig. 3D).

### DDX39B modulates FUT3 expression by regulating mRNA splicing and export

**Fig. 4:**
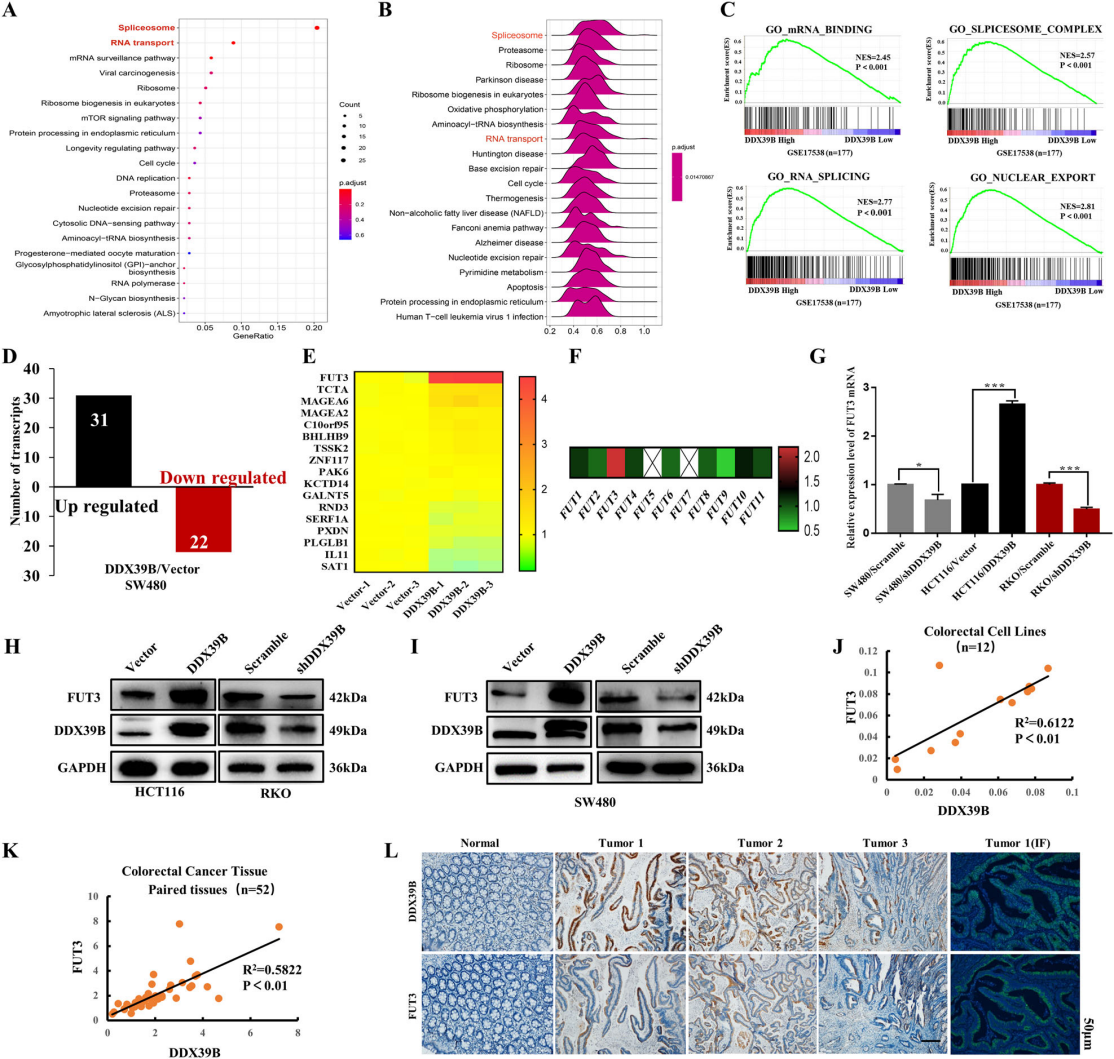
DDX39B upregulates and correlates to FUT3 expression. **A**, **B** Enrichment analysis using GSEA and GO showed RNA export and splice in CRC were correlated with DDX39B expression. **C** Relationship of certain RNA processes with low/high DDX39B expression was shown using GSE17538 (*n* = 177). **D** RNA-seq results showed upregulated and downregulated transcripts with DDX39B overexpression. **E** qRT-PCR was conducted to validate the fold changes in several gene expressions (tumor-related) in SW480/Vector and SW480/DDX39B groups. **F** Fold changes of FUT expression with DDX39B overexpression (RNA-seq data). **G**–**I** Expression levels of FUT3 mRNA and protein in CRC cells with DDX39B overexpression or knockdown; error bars, SD. **J**, **K** Correlation between DDX39B and FUT3 mRNA expression in 12 CRC/normal colon cells and in 52 paired CRC tissues. **L** IHC and IF assay using serial sections of paired CRC tissues were performed to analyze the correlation between FUT3 and DDX39B expression; scale bars, 50 μm.

Gene set enrichment analysis (GSEA) showed that DDX39B is closely associated with mRNA binding, RNA splicing, and nuclear export (Fig. 4B, C). Similar results were found in an enrichment analysis of GO—Cellular components (Fig. 4A and S2C)^26^. The RNA-sequencing (RNA-seq) results revealed altered gene expression levels in the SW480/DDX39B group (Fig. 4D and Supplementary sheet 1); then qRT-PCR was conducted to validate changes in mRNA levels. As shown in Fig. 4E, the mRNA expression level of FUT3 was significantly altered by DDX39B upregulation. Similar results were observed in HCT116 cells with DDX39B overexpression, and RKO and SW480 cells with DDX39B silencing (Fig. 4G).

Next, we explored the correlation between DDX39B and FUT3. IHC of serial CRC tissue sections (3/10 paired) showed that the change in FUT3 expression may be correlated with altered DDX39B expression (Fig. 4L). In addition, the DDX39B mRNA expression level was positively correlated with the FUT3 mRNA expression level in both paired CRC tissues and cell lines (Fig. 4J, K).

**Fig. 5:**
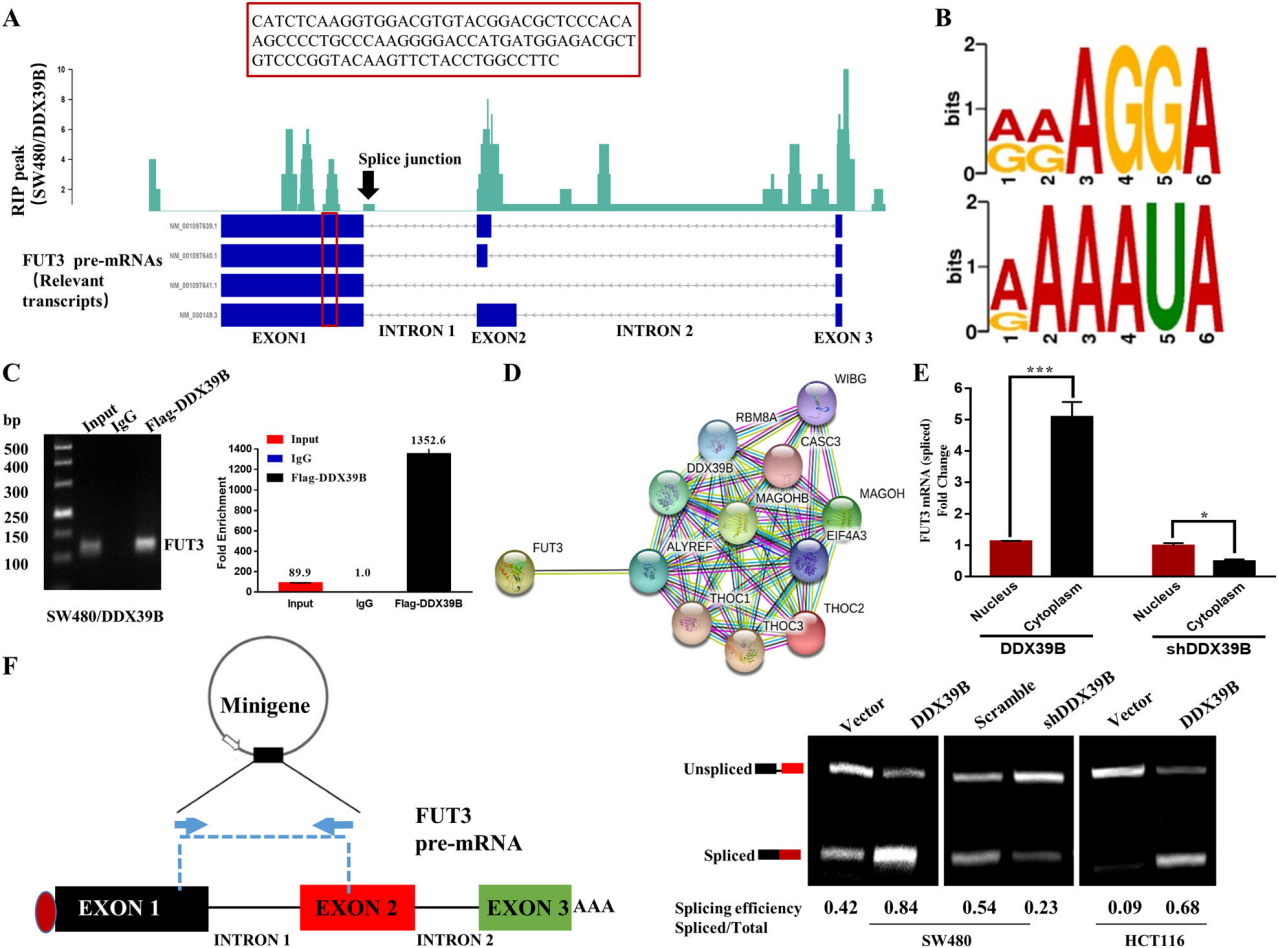
DDX39B promotes the splicing and export of FUT3 via directly binding to FUT3 pre-mRNA. **A** RIP-seq of SW480/DDX39B showed that DDX39B binds directly to multiple sites of FUT3 pre-mRNA (red box: a partial sequence of the exon1, validated by RIP). **B** The DDX39B-binding motif, presented by RIP-seq. **C** DDX39B binds the first exon of FUT3 mRNA, validated by RIP (red box in (A)); error bars, SD. **D** DDX39B interacting with FUT3 as a part of EJC/TREX complex, shown using STRING. **E** Fold change of FUT3 mRNA expression in SW480 cells with DDX39B overexpression and silencing (nuclear and cytoplasmic separation assay); error bars, SD. **F** Minigene assay showed the splicing effect of DDX39B on FUT3 pre-mRNA.

To further investigate the direct role of DDX39B in regulating the expression of FUT3, RNA-binding protein immunoprecipitation-sequencing (RIP-seq) was performed. As shown in Fig. 5A, DDX39B binds directly to multiple sites of FUT3 pre-mRNA. The DDX39B–binding motif was found to be enriched with AG (Fig. 5B). Then, we performed RIP to confirm that DDX39B could bind to the first exon of FUT3 and confirmed the binding sequence by DNA sequencing (Fig. 5A, C and S2D). Next, we analyzed the mutual effects of DDX39B and FUT3 using a network dataset from the STRING database, which showed that DDX39B, tether with ALYREF, may recruit a complex such as EJC (MAGOH/MAGOHB/EIF4A3) or TREX (THOC1-3) to affect FUT3 (Fig. 5D). A nuclear and cytoplasmic RNA separation assay demonstrated that the fold change of FUT3 mRNA expression was higher in the cytoplasm than in the nucleus (Fig. 5E), which suggests that DDX39B enhanced the export of FUT3 mRNA. Furthermore, RIP-seq also detected that DDX39B binds to the splicing site of FUT3 pre-mRNA, so we conducted a FUT3 minigene assay to validate the splicing effect of DDX39B. Semi-quantitative RT-PCR was performed to assess the unspliced and spliced FUT3 mRNA of the minigene. As shown in Fig. 5F, mature FUT3 mRNA (spliced) was significantly increased in SW480 and HCT116 cells with DDX39B overexpression and was significantly decreased in SW480 cells with DDX39B knockdown. Similar results were obtained in 293T cells with DDX39B overexpression or silencing (Fig. S2E).

Additionally, RIP-seq revealed that DDX39B could bind to the four transcripts of FUT3 and that these four transcripts were translated into the same, fully functional FUT3 protein (Ensemble online website). However, RNA-seq results detected only the increased FUT3 transcript NM_000149.4 (not shown), and thus, we assumed that DDX39B may regulate the alternative splicing of FUT3. At the splicing level, qRT-PCR using primers in exon1 (all transcripts own) and complete exon2 (only transcript NM_000149.4 owns) revealed that there are more increases of FUT3 mRNA level in products containing a longer exon2. Therefore, DDX39B may favor the inclusion of a longer FUT3 exon2 (Fig. S5).

**Fig. 6:**
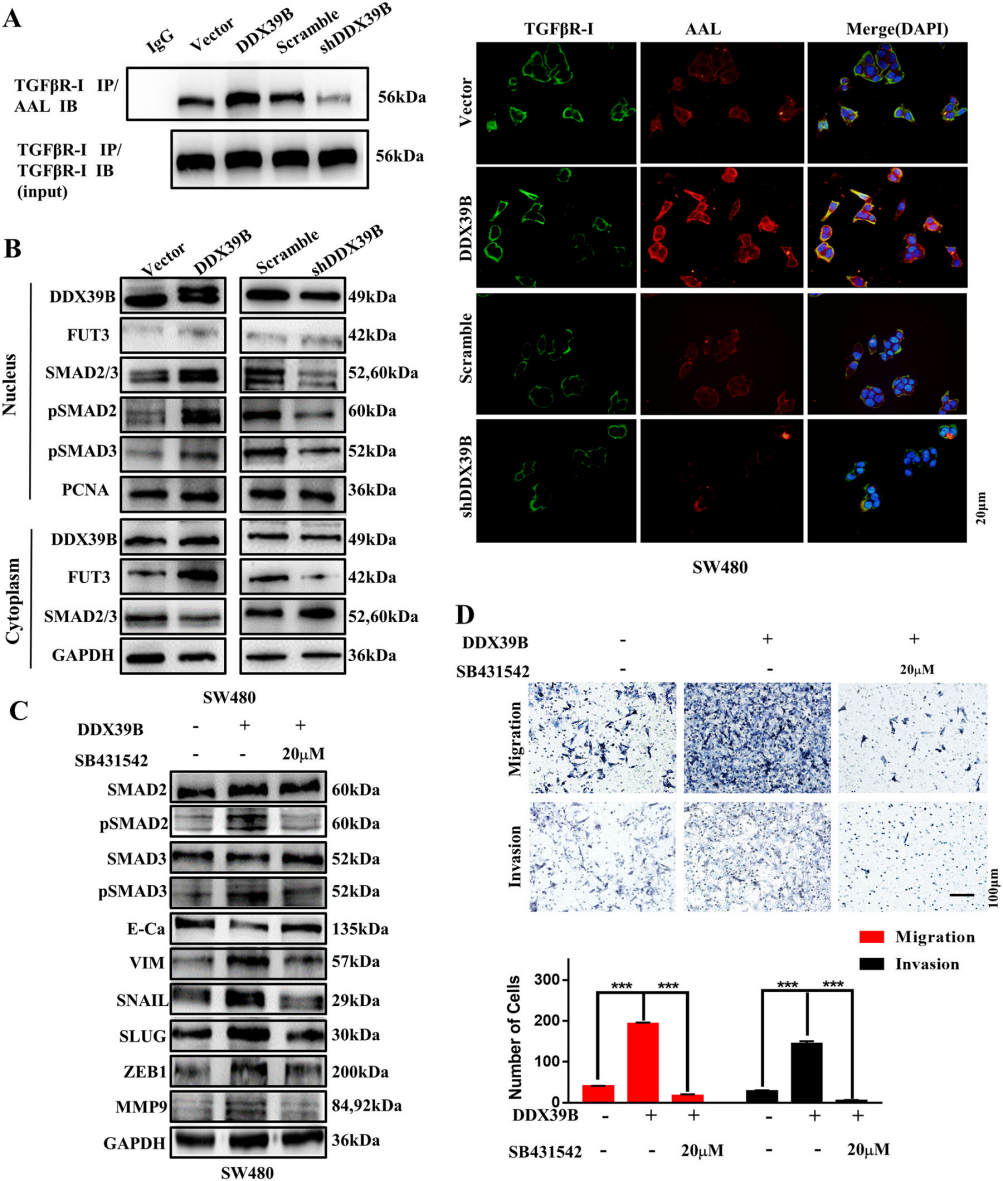
DDX39B enhances the invasive property of CRC cell via FUT3-induced L-fucosylation of TGFβR-I and then activation of the TGFβ/SMAD signaling pathway. **A** Total and fucosylated TGFβR-I (AAL) expression in SW480 cells with DDX39B overexpression and silencing; scale bars, 20 μm. **B** Targeted genes of the TGFβ signaling pathway and FUT3 expression in SW480 cells with DDX39B overexpression and silencing (nuclear and cytoplasmic separation assay). **C** Targeted genes of the TGFβ signaling pathway and EMT biomarker expressions in SW480/Vector and SW480/DDX39B cells with SB431542 (0/20 μM) treated. **D** Migration and invasion abilities in SW480/Vector and SW480/DDX39B cells with SB431542 treated (Transwell assay); error bars, SD; scale bars, 100 μm.

Then the expression levels of FUT3 protein in the DDX39B overexpression and silencing groups were analyzed. Western blotting showed that the expression levels of total FUT3 protein were increased in HCT116/DDX39B and SW480/DDX39B groups compared with HCT116/Vector and SW480/Vector groups, while the opposite effect was observed in the DDX39B silencing group (Fig. 4H, I). Moreover, a nuclear and cytoplasmic separation assay suggested that the expression changes of FUT3 protein were primarily shown in the cytoplasm, while the expression alterations of DDX39B protein were mainly in the nucleus (Fig. 6B). Similar results were obtained in the IF assay (Fig. S3D).

Thus, we explored the function of FUT3 in CRC cells. As shown in Figure S3A, FUT3 was highly expressed in the majority of gastrointestinal tumors (TCGA database online website GEPIA^27^). Moreover, Transwell and wound healing assay demonstrated that FUT3 silencing could inhibit the migration and invasion of CRC cells (Fig. S3B). Western blotting showed that FUT3 silencing might downregulate the expression of EMT marker genes, SMADs, and MMPs (Fig. S3C).

### DDX39B enhances the invasive property of CRC cell via FUT3-induced L-fucosylation of the TGFβR-I and then activation of TGFβ/SMAD signaling pathway

Lectin blotting analysis showed decreased L-fucosylation of TGFβR-I in SW480 cells with DDX39B knockdown and increased L-fucosylation of TGFβR-I in SW480 cells with DDX39B overexpression (Fig. 6A). Co-staining for TGFβR-I and AAL showed that TGFβR-I levels remained constant in SW480 cells upon DDX39B overexpression or silencing, while AAL levels were altered (Fig. S4A).

Therefore, we analyzed changes in the expression of several important genes in the TGFβ signaling pathway. Nuclear and cytoplasmic protein separation assays showed that DDX39B silencing inhibits SMAD2 and SMAD3 phosphorylation and nuclear localization, while DDX39B overexpression led to the opposite effects (Fig. 6B). Furthermore, SW480/DDX39B cells were treated with a TGFβ signaling pathway inhibitor (SB431542); western blotting and Transwell assays showed that SB431542 abrogated the effect of DDX39B in enhancing the TGFβ signaling pathway and reduced the effect of DDX39B in facilitating CRC metastasis (Fig. 6C, D).

**Fig. 7:**
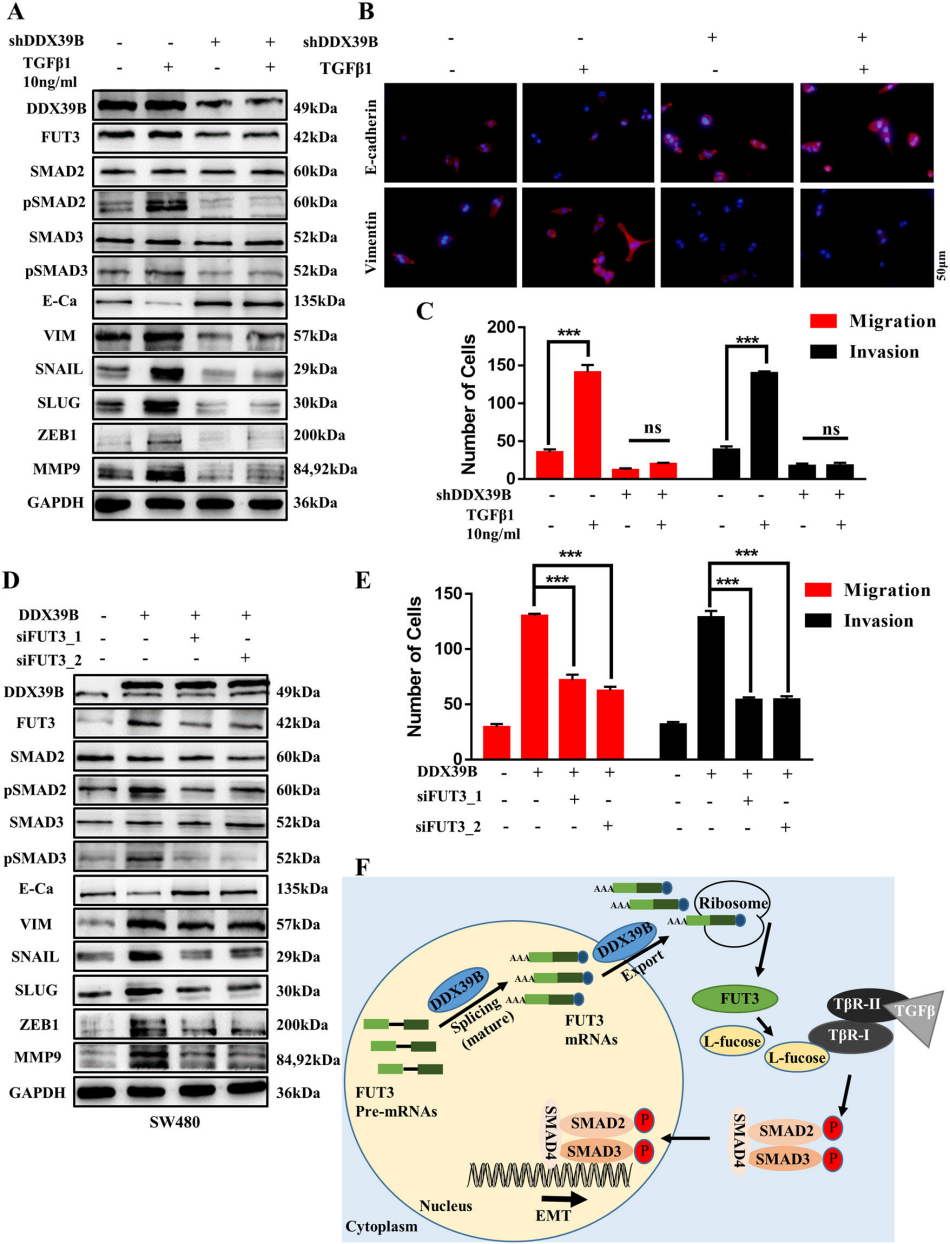
The DDX39B–FUT3–TGFβR-I axis promotes CRC metastasis and EMT. **A** Targeted genes of the TGFβ signaling pathway, EMT biomarkers and FUT3 expression in SW480/Scramble and SW480/shDDX39B cells with TGFβ1 treated (10 ng/ml). **B** E-cadherin and Vimentin expression in SW480/Scramble and SW480/shDDX39B cells with TGFβ1 treated were detected using IF; scale bars, 50 μm. **C** Migration and invasion abilities in SW480/Scramble and SW480/shDDX39B cells with TGFβ1 treated (Transwell assay); error bars, SD. **D** Targeted genes of TGFβ signaling pathway, EMT marker genes and FUT3 expression in SW480/Vector and SW480/DDX39B cells with FUT3 silencing. **E** Migration and invasion abilities in SW480/Vector and SW480/DDX39B cells with FUT3 knockdown (Transwell assay); error bars, SD. **F** Hypothesized molecular mechanism of DDX39B in the development of CRC.

Next, we assessed whether cells in the SW480/shDDX39B group were responsive to TGFβ1. TGFβ1 did not induce the expression of downstream SMADs and EMT marker genes in the SW480/shDDX39B group, but rather, it triggered the expression of SMADs and EMT marker genes in the SW480/Scramble group (Fig. 7A). SW480/Scramble cells exhibited increased migration and invasion after TGFβ1 treatment compared with SW480/shDDX39B cells (Fig. 7C and Fig. S4B). Consistent with these findings, IF was used to detect E-cadherin and Vimentin, and demonstrated that TGFβ1 did not elicit the expression of EMT marker genes in SW480/shDDX39B cells but did induce the expression of these proteins in SW480/Scramble cells (Fig. 7B).

Then, we analyzed the capacity of FUT3 to enhance DDX39B-mediated activation of the TGFβ/SMAD2 signaling pathway. Western blotting and Transwell assays revealed that the suppression of FUT3 by siRNA could partly alleviate the effect of DDX39B upregulation on TGFβ/SMAD2 pathway activation (Fig. 7D, E and Fig. S4C). Similarly, western blotting and Transwell assays revealed that FUT3 overexpression in SW480/shDDX39B cells could restore activation of the TGFβ/SMAD2 pathway (Fig. S4D, E).

## Discussion

The DDX family is implicated in many steps of RNA metabolism, and the exact function of each DDX is determined by its environment or binding partners. DDX39B is localized within nuclear speckles where most mRNA transcription and splicing occur^28^, so its core obligation is mRNA splicing and export, which may influence later steps of gene expression. Therefore, deregulation of DDX39B may exert deleterious effects on mRNA splicing/export, thus influencing the expression and function of key proteins. Although the functions of DDXs in cancers have recently been highly examined, the exact contribution of DDX39B to CRC has not yet been investigated. Our data suggested that DDX39B overexpression in CRC promoted CRC aggravation, which indicated that DDX39B may act as a tumor promoter in the development of CRC and thus needs to be thoroughly characterized.

Therefore, we sought to determine how abnormal expression of DDX39B disrupts cellular function and enhances CRC survival. Considering that protein-protein interactions are essential for RNA helicases to carry out their functions, we assumed that DDX39B exerts effects on the downstream by forming certain complexes. DDX39B was reported to be bound to mRNA during splicing, and DDX39B is retained on spliced mRNA on the exon as part of the EJC or TREX^28^. Specifically, RNA nuclear export is coupled to RNA splicing, and in one model, once DDX39B, a splicing factor, helps U2 snRNP attach to the splicing branch point, DDX39B tethers with ALYREF and other components to recruit the EJC or TREX complex and then bind to the first exon of mRNA for export^29^. In our study, RIP-seq results showed that DDX39B could bind directly to the splicing site of FUT3 pre-mRNA and that the DDX39B–binding motif is enriched with AG. 5’GU…AG-OH-3’ is known as the splicing junction, and DDX39B usually binds the 3’ AG site as a part of U2 snRNP. Moreover, the FUT3 minigene assay also validated the splicing effect of DDX39B. As mRNA export is coupled to RNA splicing, we performed RIP, cytoplasmic and nuclear RNA separation, and STRING analysis to verify that DDX39B could bind the first exon of FUT3 mRNA as a part of the mRNA export complex, such as the EJC (MAGOH/MAGOHB) or TREX (THOC1-3) complex, to promote FUT3 mRNA export. Moreover, we observed that DDX39B also binds to exon2 and may favor the longer FUT3 mRNA products that contain the complete and longer exon2, and we assumed that the longer mRNA products may be more stable. Further investigation is required to clarify how DDX39B participates in the alternative splicing of FUT3 mRNA.

Protein glycosylation is an important type of post-translational modification. Substantial data have revealed that abnormal glycosylation due to the aberrant expression of glycosylation transferases is closely correlated with tumor progression and metastasis^30–32^. FUT3, a type of fucosyltransferases (FUTs), adds L-fucose as a (1,4) linkage to GlcNAc residues in sialylated precursors to synthesize CA199 or adds L-fucose as a (1,3) linkage to GlcNAc residues in other sialylated precursors^33,34^. A previous study demonstrated that abnormal expression of FUT3 contributes to aberrant fucosylation of TGFβR-I, which eventually causes abnormal activation of the TGFβ signaling pathway^35^. Our study demonstrated that FUT3 is overexpressed in CRC and is regulated by DDX39B. Additionally, our results showed inefficient fucosylation of TGFβR-I with DDX39B knockdown, while DDX39B overexpression resulted in the opposite effect. Aberrant fucosylation of TGFβRs induced by FUT6/FUT8 has also been observed in some studies^35,36^. Our data (RNA-seq) showed that DDX39B does not modulate the expression of FUTs other than FUT3.

The TGFβ signaling pathway is an important cellular pathway that regulates many processes and plays a significant role in cellular proliferation, differentiation, and apoptosis. TGFβR-I is an essential component of the canonical TGFβ signaling pathway, and alterations in this protein could affect activation of the TGFβ signaling pathway. In our study, DDX39B intervened in the TGFβ/SMAD2 signaling pathway by upregulating FUT3 and catalyzing the fucosylation of TGFβR-I, after which the activated TGFβ/SMAD2 signaling pathway upregulated the expression of MMPs and EMT transcriptional factors, such as Snail, Slug, and ZEB1. MMPs can degrade basement membrane components to promote tumor cell infiltration of the blood. Together with EMT, a decrease in epithelial marker (E-cadherin) and an increase in mesenchymal marker (Vimentin) expression drive the reduced adhesion of cells and loss of cell polarity and eventually contribute to CRC progression.

Our study was primarily focused on the mechanism of DDX39B in the progression of CRC, and thus, our study was limited by the lack of large clinical samples.

In conclusion, DDX39B acts as a tumor promoter in CRC by upregulating the expression of FUT3 and then promoting the fucosylation of TGFβR-I, which subsequently enhances activation of the TGFβ/SMAD2 signaling pathway to facilitate the invasion and metastasis of CRC. Taken together, our findings regarding the DDX39B–FUT3–TGFβR-I axis might provide new insight into CRC tumorigenesis and suggest that the proteins in this axis can serve as molecular markers for CRC detection or treatment.

## Materials and methods

### Clinical specimens

Human CRC tissues and adjacent normal colon tissues used in our study were obtained from CRC patients who underwent surgical resection at Nanfang Hospital of Southern Medical University. A diagnosis of CRC was confirmed histopathologically for each sample and none of these patients had received chemotherapy or radiotherapy before surgery. The protocols used in this study were approved by Nanfang Hospital’s Protection of Human Subjects Committee.

### Immunohistochemistry (IHC) and hematoxylin and eosin (HE) staining

See Supplementary materials and methods for details.

### Cell culture

Human CRC cell lines (SW480, SW620, HT29, HCT116, RKO, LoVo, 293T) were purchased from the Cell Bank of Type Culture Collection (CBTCC, China Academy of Sciences, Shanghai, China). All cells were cultured in Dulbecco’s modified Eagle’s medium (DMEM) (Gibco, Carlsbad, CA) supplemented with 10% fetal bovine serum (FBS; Gibco, Carlsbad, CA) at 37 °C with a humidity of 5% CO_2_. For activation/inactivation of the TGFβ signaling pathway, CRC cells were treated with TGFβ1 (10 ng/ml, Peprotech, Rocky Hill, NJ, USA) or SB431542 (20 μM, Selleck Chemicals, Houston, TX, USA).

### Small interfering RNA, plasmid, and lentivirus transfection

See Supplementary materials and methods for details.

### Western blotting and lectin blotting analyses

See Supplementary materials and methods for details.

### Total, cytoplasmic and nuclear RNA isolation and quantitative real-time PCR (qRT-PCR)

See Supplementary materials and methods for details.

### Migration and invasion assays

See Supplementary materials and methods for details.

### Immunofluorescence

See Supplementary materials and methods for details.

### RNA-sequencing (RNA-seq)

We sent SW480/Vector and SW480/DDX39B cells to BGI China. Total RNA was extracted using TRIzol reagent (Invitrogen, Carlsbad, CA, USA). After the total RNA was qualified and quantified, oligo(dT)-attached magnetic beads were used to purify the mRNA. They were sequenced and generated on the BGIseq500 platform (BGI, Shenzhen, China). RNA-seq results were visualized with the Broad Institute’s Integrative Genomic Viewer.

### RNA-binding protein immunoprecipitation(RIP)

A Magna RIP Kit (Billerica MA, USA, No.17-701) was used for RIP. In all, 2 × 10^7^ SW480/DDX39B cells (per immunoprecipitation) were collected first, according to the detailed specifications of the Magna RIP Kit. Ten microliters of antibody against the DYKDDDDK Tag (#66008–3, Proteintech) was used for RIP.

### RNA-binding protein immunoprecipitation-sequencing

We used a Magna RIP Kit (17-700) to carry out RIP experiments on SW480/DDX39B cells, and sent RIP and Input products to RIBO, China. RIP and input products passed quality-control tests and were sequenced on an Illumina platform. The RIP-seq report was presented by Guangzhou RIBO Biotechnology Co., Ltd.

### Minigene assay

Human FUT3 intron 1 with 162 bp of flanking exon1 and 124 bp of flanking exon2 was constructed in vitro and cloned into a minigene pcDNA3.1 construct (Hanyi Tech. Guangdong, China). The FUT3 minigene was transfected into CRC and 293T cells with DDX39B overexpression and silencing. After 24 h, the cells were harvested and subjected to RNA isolation, reverse transcription, and PCR analysis (Takara RR901A procedures). Subsequently, PCR products were separated by a 2% agarose gel. Image J was applied to quantify the unspliced and spliced minigene bands.

### Tumorigenesis in nude mice

See Supplementary materials and methods for details.

### Statistical analyses

Statistical analyses were performed using Graph-Pad Prism software 6.0 (GraphPad Software, San Diego, CA, USA) and Microsoft Excel 2016 (Microsoft, Redmond, WA, USA). Student’s t test or nonparametric ANOVA was applied for comparisons between groups. Correlation analysis was assessed by determining the Spearman’s rank correlation coefficient. Data are expressed as mean ± SD (*P < 0.05, **P < 0.01, ***P < 0.001, ****P < 0.0001 indicated significance; ns = not significant).

## Supplementary information

Supplementary Figure 1

Supplementary Figure 2

Supplementary Figure 3

Supplementary Figure 4

Supplementary Figure 5

Supplementary Figure 6

Supplementary Figure 7

Supplementary Figure 8

Supplementary Figure 9

Supplementary Figure Legends

Supplementary Materials and Methods

Supplementary Table 1

Supplementary Table 2

Supplementary sheet1

## Data Availability

All data generated and analyzed during this study are included in this manuscript and supplementary information files.
