# Supplementary figures and images for: The DDX39B/FUT3/TGFβR-I axis promotes tumor metastasis and EMT in colorectal cancer
Source: Cell Death Dis. 2021 Jan 12;12(1):74. doi: 10.1038/s41419-020-03360-6 (PMC7803960; doi:10.1038/s41419-020-03360-6)

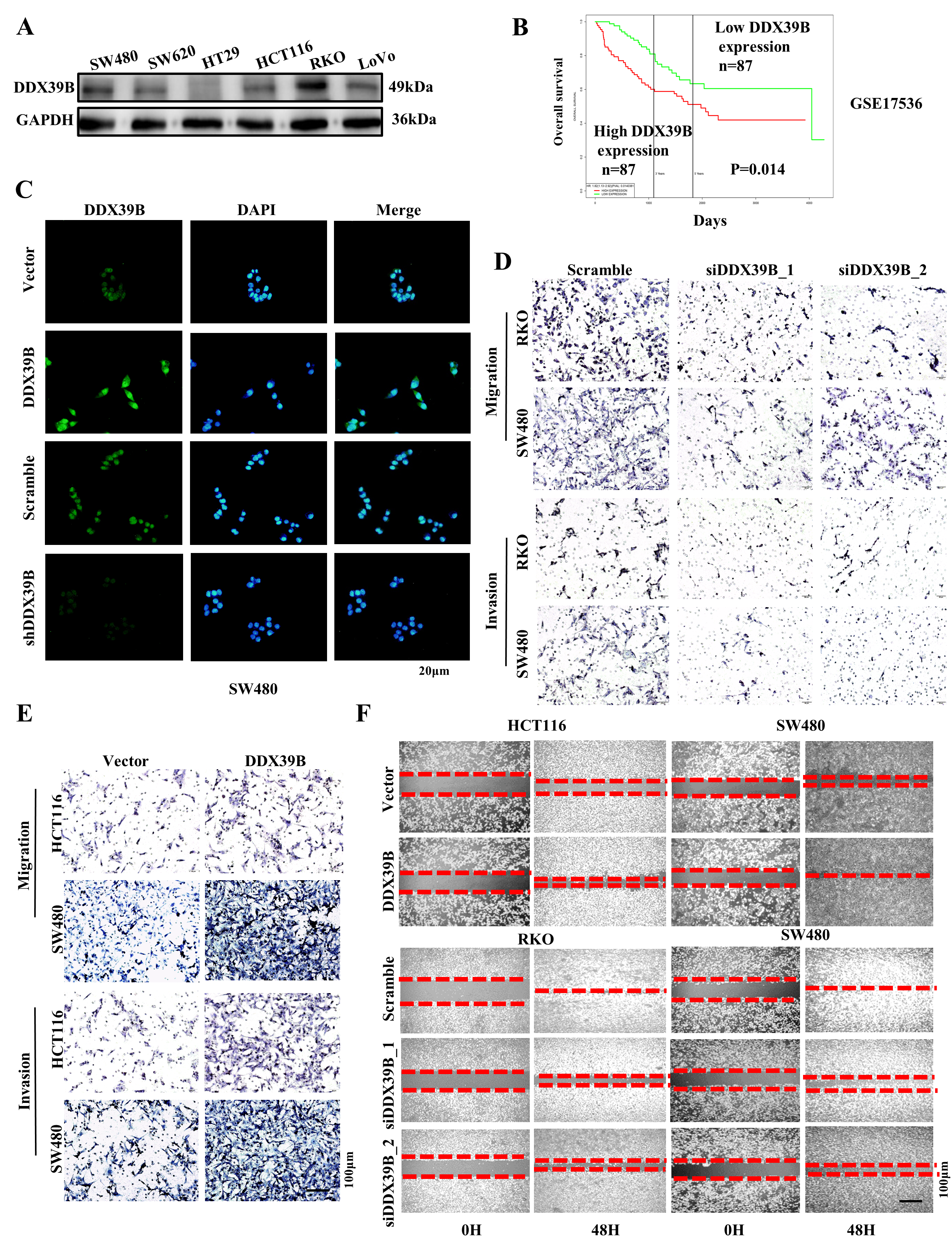

Supplement: Supplementary file 1 — Supplementary Figure 1 [file 41419_2020_3360_MOESM1_ESM.tif]

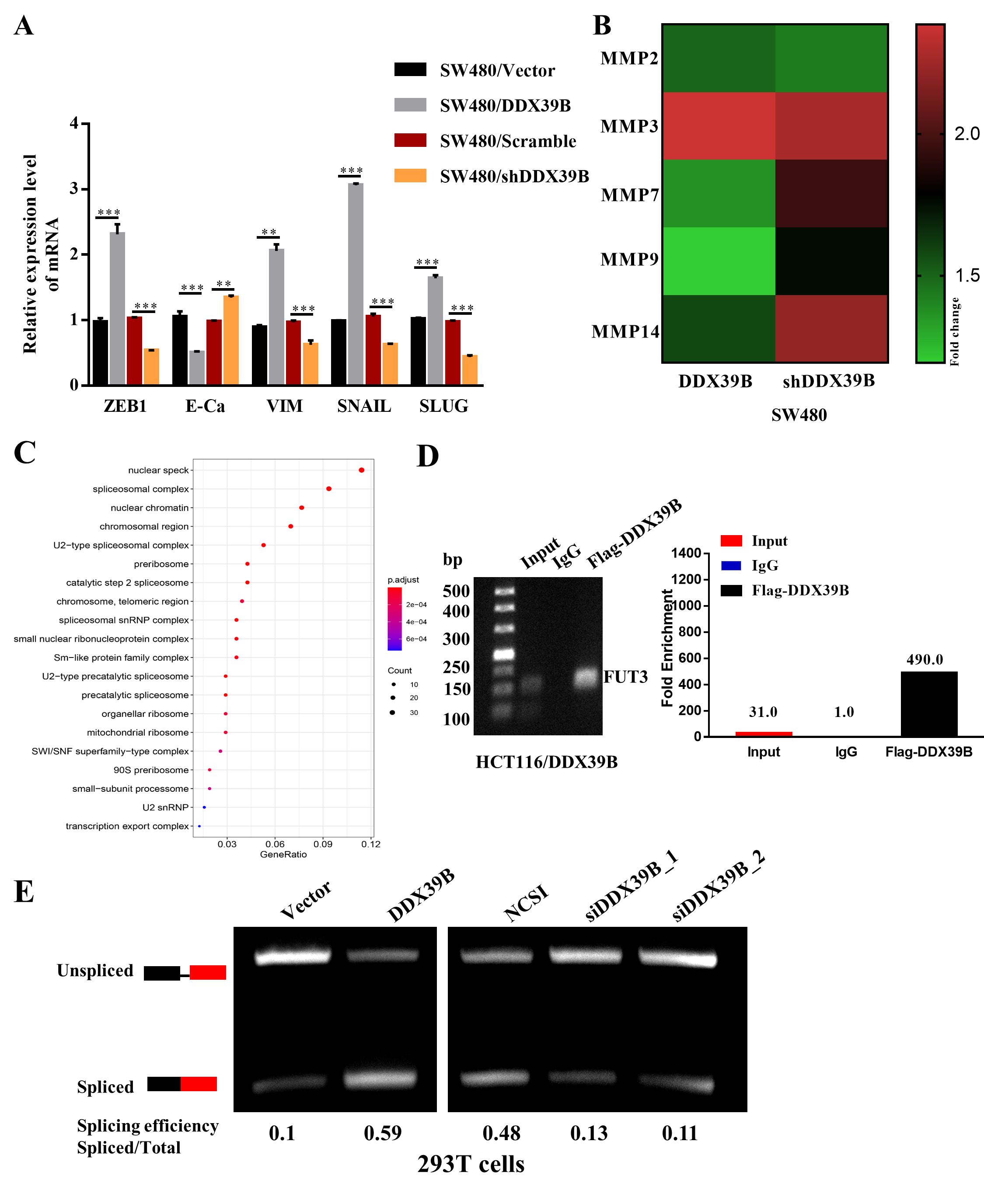

Supplement: Supplementary file 2 — Supplementary Figure 2 [file 41419_2020_3360_MOESM2_ESM.tif]

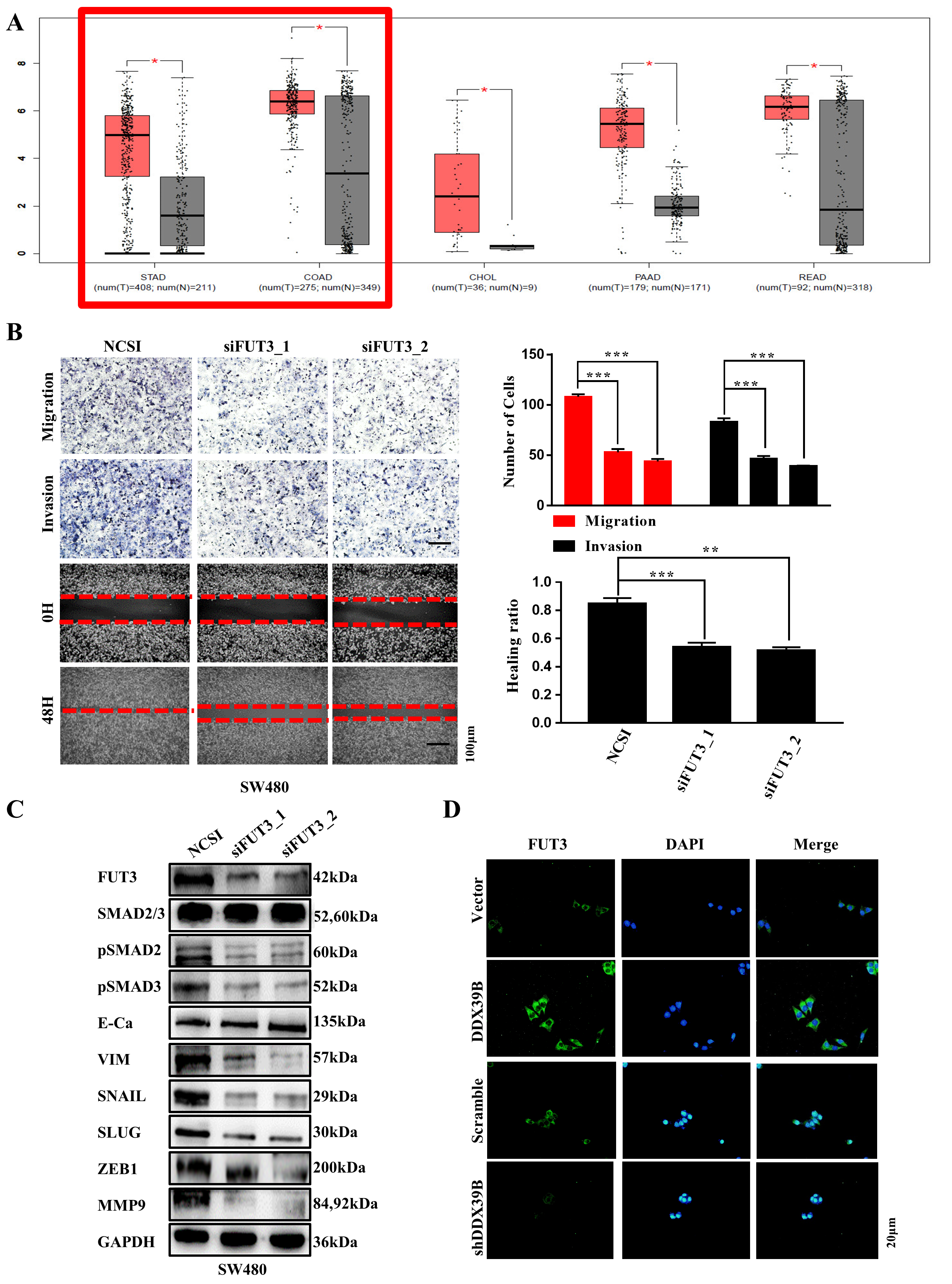

Supplement: Supplementary file 3 — Supplementary Figure 3 [file 41419_2020_3360_MOESM3_ESM.tif]

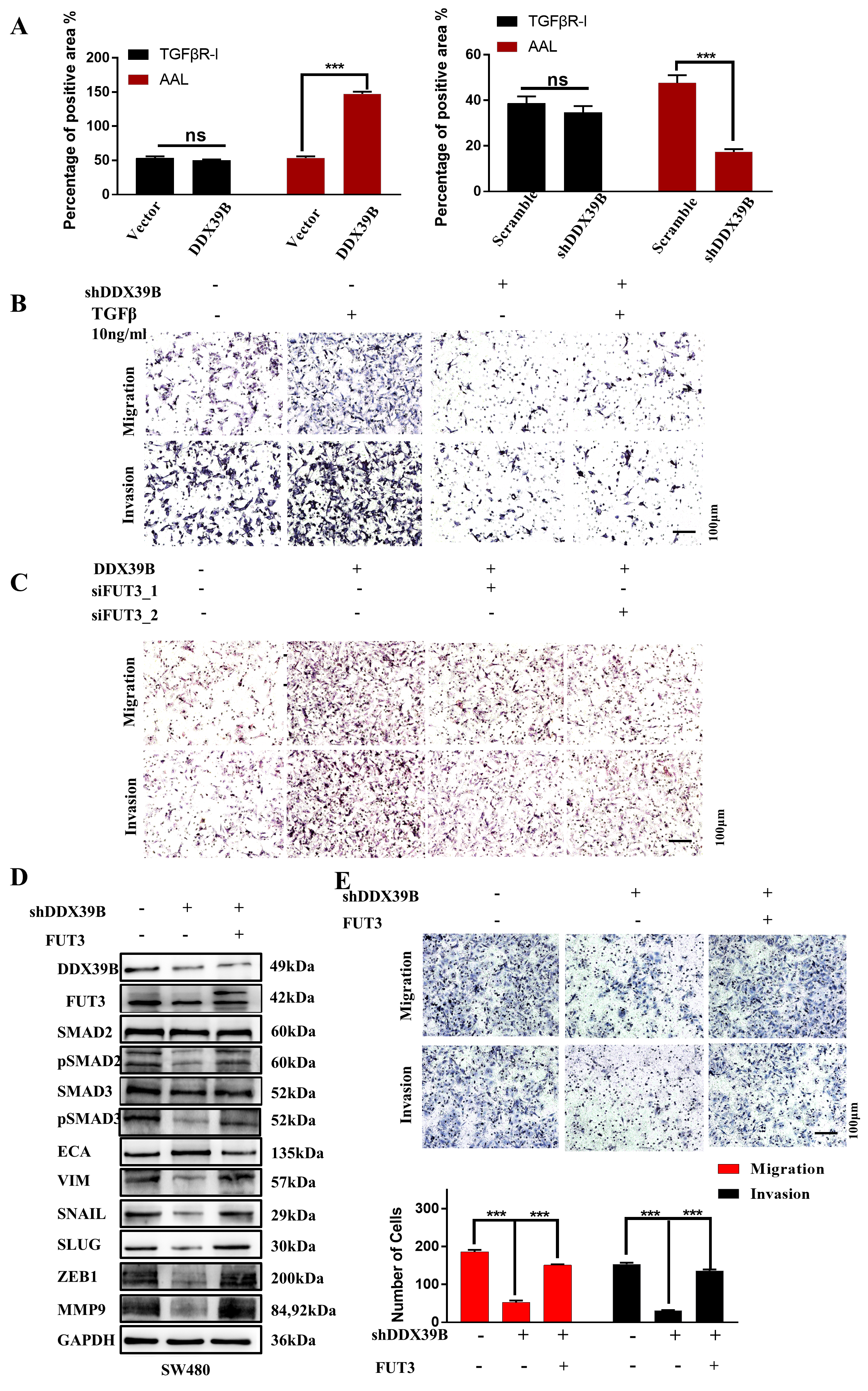

Supplement: Supplementary file 4 — Supplementary Figure 4 [file 41419_2020_3360_MOESM4_ESM.tif]

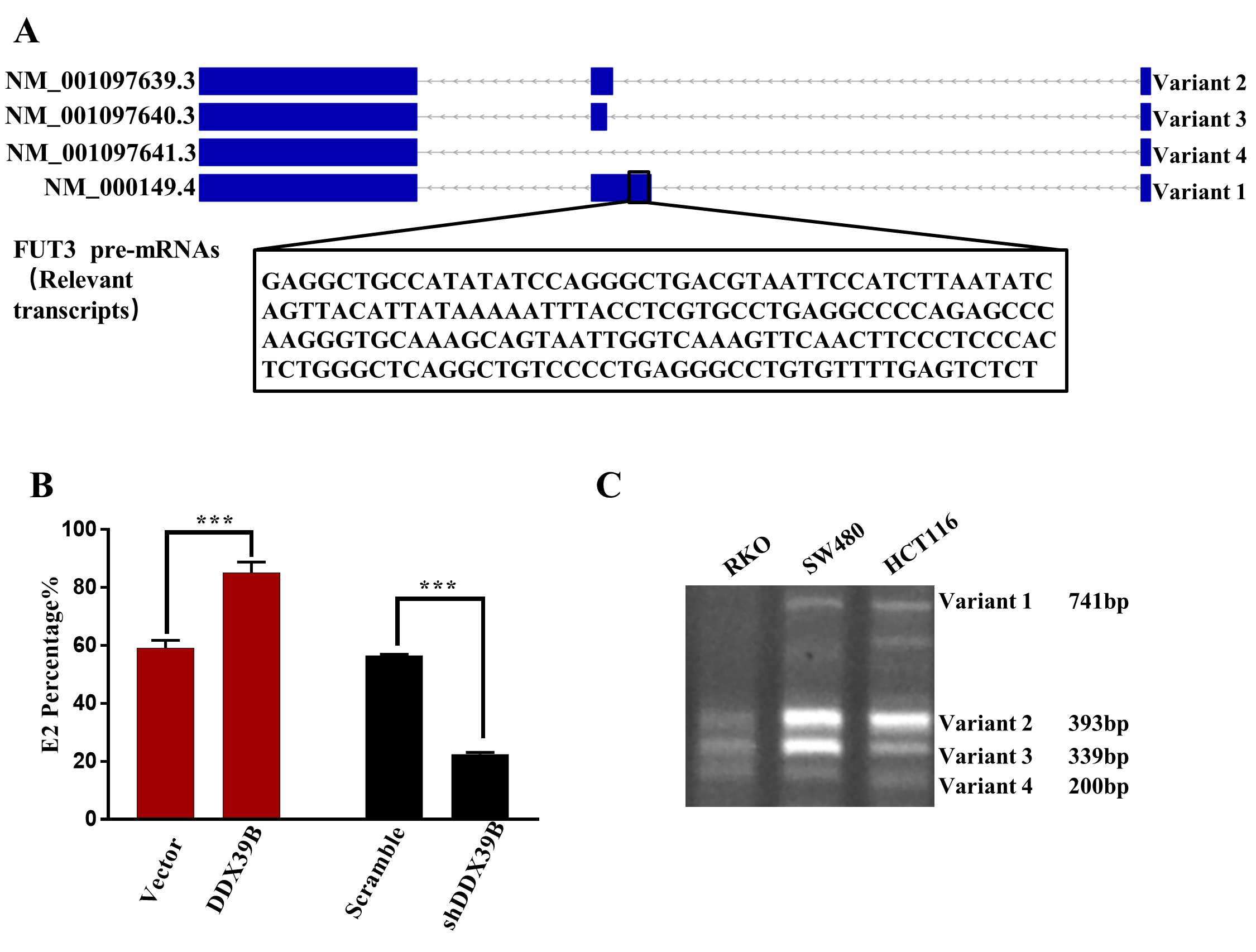

Supplement: Supplementary file 5 — Supplementary Figure 5 [file 41419_2020_3360_MOESM5_ESM.tif]

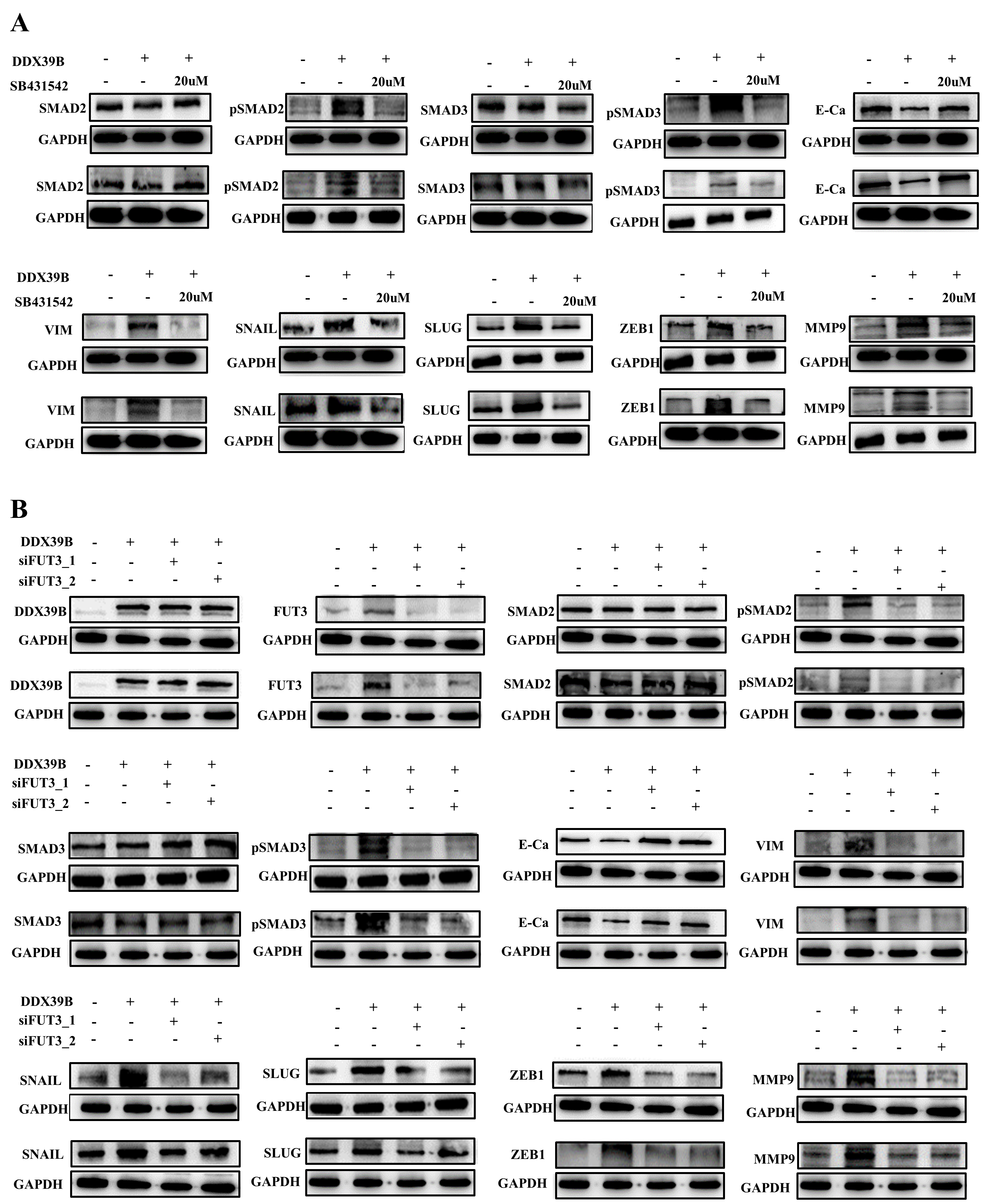

Supplement: Supplementary file 6 — Supplementary Figure 6 [file 41419_2020_3360_MOESM6_ESM.tif]

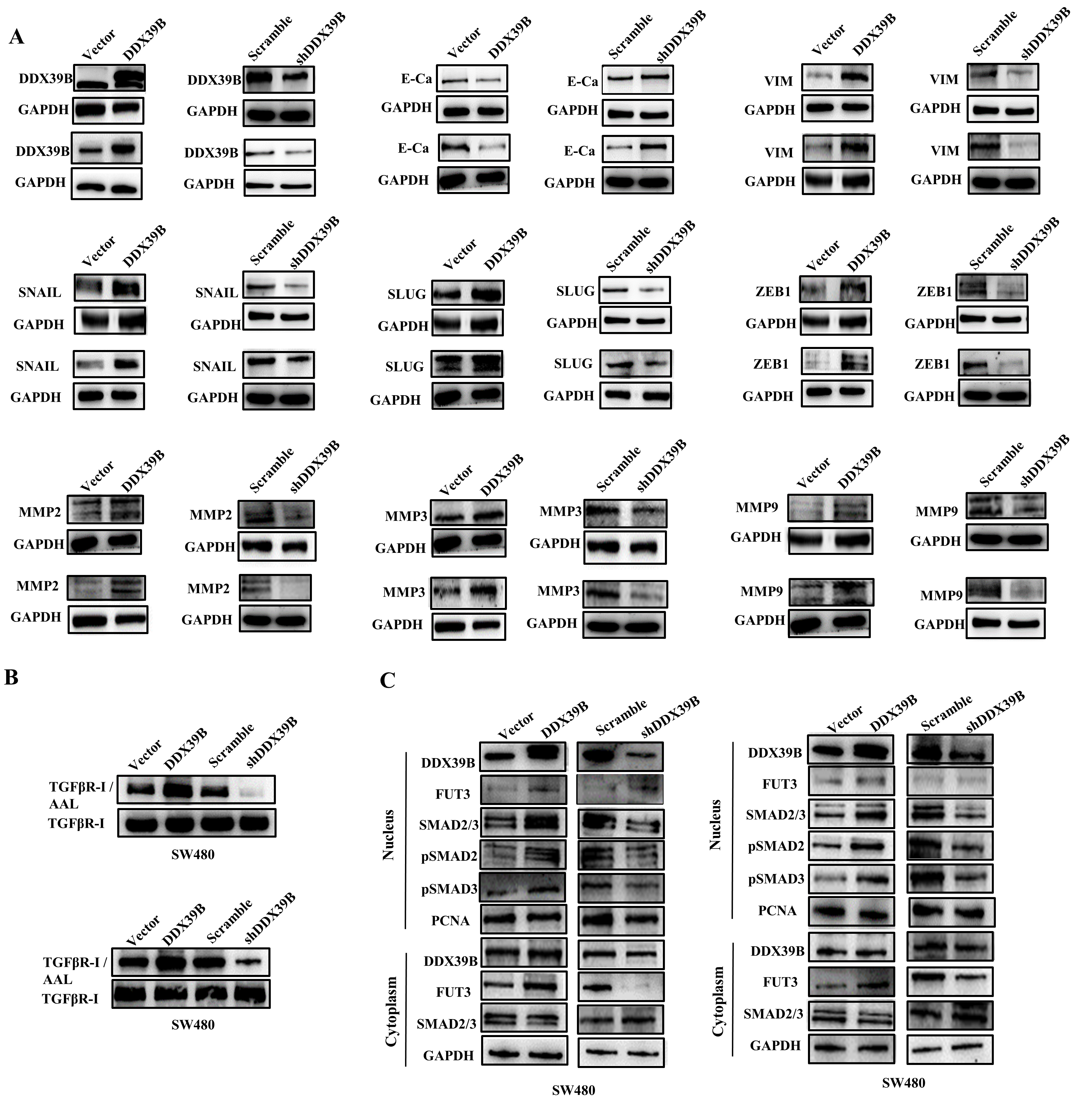

Supplement: Supplementary file 7 — Supplementary Figure 7 [file 41419_2020_3360_MOESM7_ESM.tif]

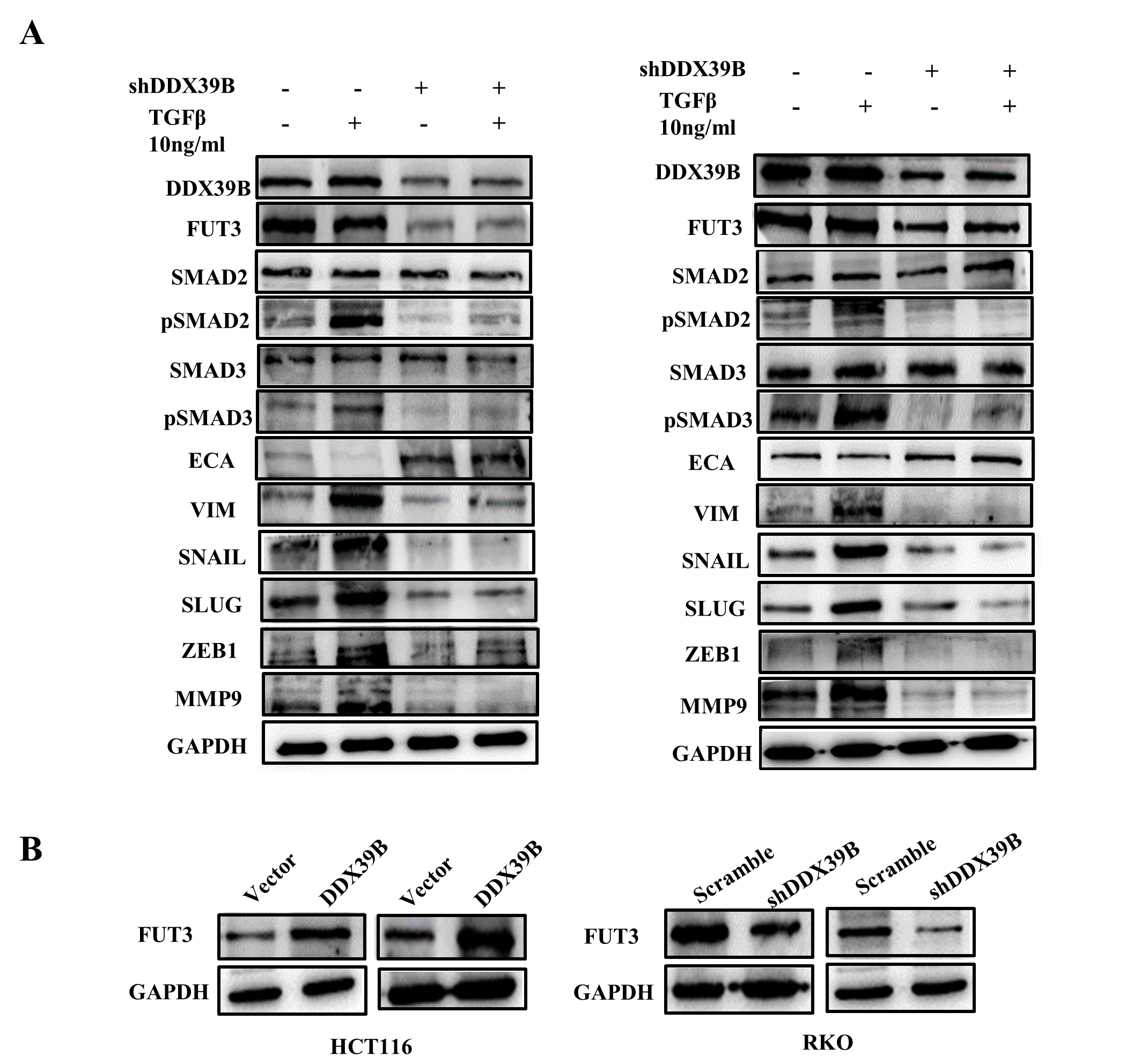

Supplement: Supplementary file 8 — Supplementary Figure 8 [file 41419_2020_3360_MOESM8_ESM.tif]

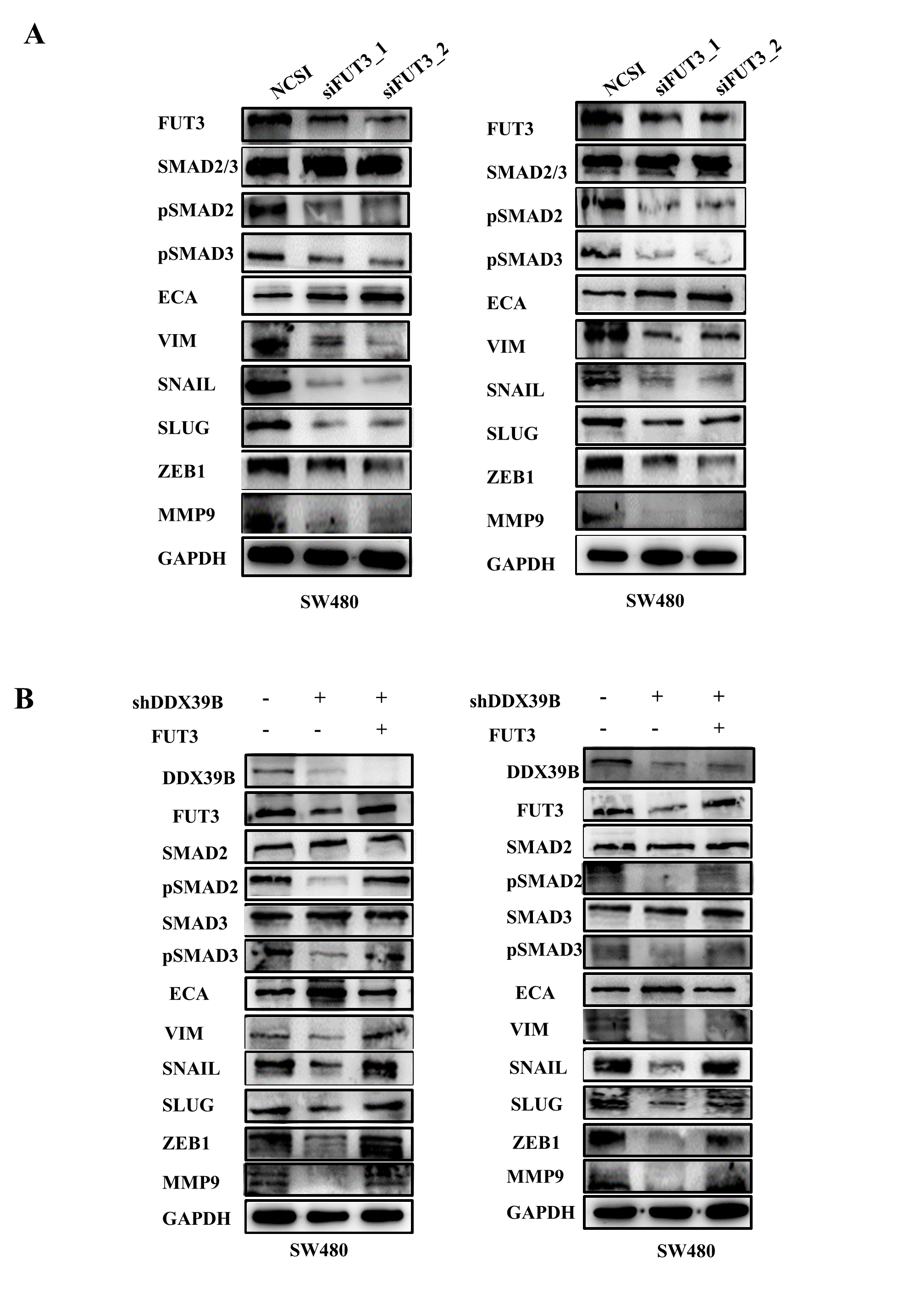

Supplement: Supplementary file 9 — Supplementary Figure 9 [file 41419_2020_3360_MOESM9_ESM.tif]
