# Supplementary material for: The DDX39B/FUT3/TGFβR-I axis promotes tumor metastasis and EMT in colorectal cancer
Source: Cell Death Dis. 2021 Jan 12;12(1):74. doi: 10.1038/s41419-020-03360-6 (PMC7803960; doi:10.1038/s41419-020-03360-6)
Supplement: Supplementary file 10 — Supplementary Figure Legends [file 41419_2020_3360_MOESM10_ESM.docx]

**S-Figure 1. A.** Expression of DDX39B protein detected by western blotting in CRC cell lines. **B.** Overall survival with low/high DDX39B expression was analyzed using GSE17536. **C.** Immunofluorescence were performed to detect DDX39B in SW480 cells with DDX39B overexpression and silencing; Scale bars, 20μm. **D.** Migration and invasion abilities were evaluated by Transwell assay in SW480 and RKO cells with DDX39B silencing; Scale bars, 100μm. **E.** Migration and invasion abilities were evaluated by Transwell assay in SW480 and HCT116 cells with DDX39B upregulation; Scale bars, 100μm. **F.** Migration capacity was assessed by Wound healing assay in HCT116, SW480 and RKO cells with DDX39B overexpression and silencing; Scale bars, 100μm.

**S-Figure 2. A.** Expression of EMT marker genes detected by qPCR in SW480 cells with DDX39B overexpression and silencing; Error bars, SD. **B.** Expression of MMPs detected by qPCR in SW480 cells with DDX39B overexpression and silencing. **C.** Enrichment analysis of GO-Cellular components in CRC with DDX39B expression. **D.** RIP results showed that DDX39B binds the first exon of FUT3 in HCT116/DDX39B cells; Error bars, SD. **E.** Minigene assay in 293T cells showed that DDX39B promotes the splicing of FUT3 pre-mRNA.

**S-Figure 3. A.** FUT3 is highly expressed in the majority of gastrointestinal tumors, shown by TCGA database online website GEPIA (<http://gepia.cancer-pku.cn/>). **B.** Migration and invasion abilities were evaluated by Transwell and Wound healing assay in SW480 cells with FUT3 knockdown; Error bars, SD; Scale bars, 100μm. **C.** Targeted genes of TGFβ signaling pathway and EMT marker genes expressions were detected by western blotting in SW480 cells with FUT3 knockdown. **D.** Immunofluorescence were performed to detect FUT3 in SW480 cells with DDX39B overexpression and silencing; Scale bars, 20μm.

**S-Figure 4. A.** Fluorescence value statistics of total and fucosylated TGFβR-I (AAL) in SW480 cells with DDX39B overexpression and silencing groups; Error bars, SD. **B.** Migration and invasion abilities were assessed by Transwell assay in SW480/Scramble and SW480/shDDX39B cells with TGFβ1 treated; Scale bars, 100μm. **C.** Migration and invasion abilities were assessed by Transwell assay in SW480/Vector and SW480/DDX39B cells with FUT3 silencing; Scale bars, 100μm. **D.** Expression of FUT3, targeted genes of TGFβ signaling pathway and EMT marker genes were detected by western blotting in SW480/Scramble and SW480/shDDX39B cells with FUT3 overexpression. **E.** Migration and invasion abilities were assessed by Transwell assay in SW480/Scramble and SW480/shDDX39B cells with FUT3 overexpression; Error bars, SD; Scale bars, 100μm.

**S-Figure 5. A.** FUT3 splicing variants and corresponding transcripts. **B.** The expression percentage of FUT3 splicing variant 1, detected by qRT-PCR using different primers; Error bars, SD. **C.** Agarose gel electrophoresis of RT-PCR products showed the FUT3 splicing variants in CRC cell lines.

**S-Figure 6. A.** Independent repeated experiments on targeted genes of TGFβ signaling pathway and EMT biomarkers expressions in SW480/Vector and SW480/DDX39B cells with SB431542 (0/20μM) treated. **B.** Independent repeated experiments on targeted genes of TGFβ signaling pathway, EMT marker genes and FUT3 expressions in SW480/Vector and SW480/DDX39B cells with FUT3 silencing.

**S-Figure 7. A.** Independent repeated experiments on EMT marker genes and MMPs expressions in SW480 cells with DDX39B overexpression and silencing. **B.** Independent repeated experiments on total and fucosylated TGFβR-I expressions (AAL) in SW480 cells with DDX39B overexpression and silencing. **C.** Independent repeated experiments on targeted genes of TGFβ signaling pathway and FUT3 expressions in SW480 cells with DDX39B overexpression and silencing (Nuclear and cytoplasmic separation assay).

**S-Figure 8. A.** Independent repeated experiments on targeted genes of TGFβ signaling pathway, EMT biomarkers and FUT3 expressions in SW480/Scramble and SW480/shDDX39B cells with TGFβ1 treated (10ng/ml). **B.** Independent repeated experiments on FUT3 expressions in HCT116 and RKO cells with DDX39B overexpression or silencing.

**S-Figure 9. A.** Independent repeated experiments on targeted genes of TGFβ signaling pathway and EMT marker genes expressions in SW480 cells with FUT3 silencing. **B.** Independent repeated experiments on expression of FUT3, targeted genes of TGFβ signaling pathway and EMT marker genes in SW480/shDDX39B cells with FUT3 overexpression.
