# Supplementary material for: The DDX39B/FUT3/TGFβR-I axis promotes tumor metastasis and EMT in colorectal cancer
Source: Cell Death Dis. 2021 Jan 12;12(1):74. doi: 10.1038/s41419-020-03360-6 (PMC7803960; doi:10.1038/s41419-020-03360-6)
Supplement: Supplementary file 11 — Supplementary Materials and Methods [file 41419_2020_3360_MOESM11_ESM.docx]

**Immunohistochemistry (IHC) and hematoxylin and eosin (HE) staining**

IHC and HE staining were performed according to the manufacturer’s instructions (ZSGB-BIO, Beijing, China). For IHC, the slides were washed and subjected to antigen retrieval in a microwave in EDTA buffer (pH=9.0). Rabbit anti-DDX39B (1:100; Proteintech, Chicago, IL, USA), rabbit anti-FUT3 (1:100; Affinity Biosciences, Cincinnati, OH, USA), rabbit anti-MMP9 (1:100; Proteintech, Chicago, IL, USA), mouse anti-E-cadherin (1:3000; Cell Signaling Technology, MA, USA) and mouse anti- Vimentin (1:100; Cell Signaling Technology, MA, USA) were applied. After dehydration and mounting, gene expression was visualized by microscopy. One independent pathologist assessed and calculated the gray values for pathological scoring using Image J.

**Small interfering RNA, plasmid and lentivirus transfection**

Small interfering RNA and plasmid transfection experiments were carried out using Lipofectamine 3000 reagent (Invitrogen; Carlsbad, CA, USA). Control and DDX39B or FUT3 siRNA (GenePharma, Shanghai, China) were transfected into SW480 and RKO cells according to the manufacturer’s instructions. The FUT3 plasmid (NM_000149) was transfected into SW480/shDDX39B cells according to the same steps (Hanyi Tech. Guangdong. China). The lentivirus human DDX39B construct was generated by cloning PCR-amplified full-length DDX39B cDNA (NM_004640). The DDX39B shRNA sequence was selected from siDDX39B_2. The control, DDX39B and shRNA lentiviruses (Lianfeng Technology, Shanghai, China) were transfected into RKO, SW480 and HCT116 cells according to the manufacturers’s instructions. Then, SW480 and HCT116 cells with overexpressed DDX39B were cultured in medium containing puromycin (BioFroxx, Germany)(2μg/ml), while SW480 and RKO cells with DDX39B knockdown were cultured in medium containing blasticidin (Yesen, China)(10μg/ml). The corresponding sequences are described in Supplementary Table 2.

**Western blotting and lectin blotting analyses**

Western blotting (total and nuclear and cytoplasmic separation assay) was performed as previously described, using the following antibodies: polyclonal anti-DDX39B (1:1000), polyclonal anti-FUT3 and anti-TGFβR-I (1:1000; Affinity Biosciences, Cincinnati, OH, USA), anti-E-cadherin, anti-Vimentin, anti-MMPs, anti-SNAIL, anti-SLUG, anti-ZEB1, and anti-SMADs (1:1000; Cell Signaling Technology, Danvers, MA, USA). The loading control was a mouse anti-GAPDH monoclonal antibody (1:1000; Proteintech, Chicago, IL, USA). For the lectin blotting assay, protein lysates were prepared and incubated with an anti-TGFβR-I antibody overnight at 4℃ with rotation. Then, 20μl of magnetic proteinA/G beads was added into the above protein-antibody lysates and incubated for 2-4h at 4℃. Procedures similar to those used for western blotting were carried out. For immunoprecipitation protein, 0.5μg/ml biotinylated *Aleuia aurantia* lectin (preferentially recognizes Fuc-1,3GlcNac) in TBST was incubated with the membranes for 1h at room temperature. HRP-conjugated streptavidin was then incubated with the membranes for 30 minutes at room temperature. Both western and lectin blotting were performed at least three times for confirmation. And the average gray values of these proteins were analyzed using Image J software.

**Total, cytoplasmic and nuclear RNA isolation and quantitative real-time PCR (qRT-PCR)**

Total RNA was extracted using TRIzol reagent (TaKaRa, Osaka, Japan). Cytoplasmic and nuclear RNA isolation was performed using a Cytoplasmic&Nuclear RNA Purification Kit (Norgen, Canada). qRT-PCR was performed using a PrimeScript RT Reagent Kit (#RR036A, TaKaRa) and a SYBR Premix Ex Taq(#RR620A, TaKaRa) according to the manufacturer’s instructions. The loading control was glyceraldehyde-3-phosphate dehydrogenase (GAPDH) or 18s. Data were analyzed using the 2-∆∆Ct method. The sequences of specific primers are listed in Supplementary Table 1.

**Migration and invasion assays**

Transwell chambers equipped with 8-μm membranes (Corning Incorporated, Corning, NY,USA) were used for cell migration and invasion. Additionally, 100μl of BD Matrigel (diluted 1:8 in serum-free medium) was used to cover the membranes prior to the cell invasion assay. Cells (1×10^5^ cells/200μl) suspended in serum free medium were added to the top of the Transwell chambers, while 500μl of medium containing10% FBS was added to the bottom of the chambers. After 36-48h of incubation at 37℃, cells were harvested, fixed in 4% paraformaldehyde for 20 minutes at room temperature and then stained with hematoxylin. The membranes were mounted on glass slides, and cells in 5 random fields were counted by microscopy. For the wound healing assay, cells at 70-80% confluence were seeded in 6-well plates, and then wounded with a pipette tip at time 0. Then, the cells were cultured in DMEM, and photos were taken at 24 and 48h after scratching. The gap distance was quantified using Image J. Each experiment was repeated three times.

**Immunofluorescence**

Cells grown on coverslips were fixed in 4% paraformaldehyde, after which 0.5% Triton X-100 was applied to permeabilize the cell membrane, and 1% bovine serum albumin was used to block nonspecific binding. The coverslips were incubated overnight with primary antibodies against DDX39B (1:50), FUT3 (1:50), E-Cadherin (1:100), Vimentin (1:100), MMP9 (1:100), and *Aleuia aurantia* lectin (20μg/ml; AAL; Vector, Burlingame, USA). Then, the cells were washed with PBST and incubated with fluorescein isothiocyanate-conjugated secondary antibodies. For F-actin staining, cells on coverslips were incubated with rhodamine-conjugated phallotoxin for 1h. After the cells were washed with PBST, DAPI Fluoromount-GTM was applied for nuclear staining after which the coverslips were sealed. The coverslips were visualized under an Olympus BX40 fluorescence microscope (Olympus Optical Co., Ltd., Tokyo, Japan).

**Tumorigenesis in nude mice**

To generate the orthotopic transplantation model, SW480/Scramble and SW480/shDDX39B cells (1×10^7^) were injected into the ileocecal serosa of 4-6week old male BALB/c athymic mice (nu/nu). All mice were purchased from the Medical Animal Center of Guangdong Province (Guangzhou, China), and were housed under specific pathogen-free conditions. Animal protocols were approved by the Use Committee for Animal Care. After 8 weeks, the mice were sacrificed and their tumors were then paraffin-embedded. HE staining was performed to detect metastasis in the spleen, and the orthotopic tumors were subjected to IHC staining and immunofluorescence assay.
