# Supplementary material for: The DDX39B/FUT3/TGFβR-I axis promotes tumor metastasis and EMT in colorectal cancer
Source: Cell Death Dis. 2021 Jan 12;12(1):74. doi: 10.1038/s41419-020-03360-6 (PMC7803960; doi:10.1038/s41419-020-03360-6)
Supplement: Supplementary file 12 — Supplementary Table 1 [file 41419_2020_3360_MOESM12_ESM.docx]

**Supplementary Table 1. Primer sequences used for qPCR.**

| **Primer** | **Forward Sequence（5’to3’）** | **Reverse Sequence（5’to3’）** |
| --- | --- | --- |
| **DDX39B** | **ACTCGGGAGTTGGCTTTTCAG** | **GAGTCCCCACGACGATATGC** |
| **MMP2** | **GATACCCCTTTGACGGTAAGGA** | **CCTTCTCCCAAGGTCCATAGC** |
| **MMP3** | **GGTGTGGAGTTCCTGATGTTGGTC** | **AGCCTGGAGAATGTGAGTGGAGTC** |
| **MMP7** | **AAATGCCAACAGTTTAGAAGCC** | **ATTATTTCTATGACGCGGGAGT** |
| **MMP9** | **CAGTACCGAGAGAAAGCCTATT** | **CAGGATGTCATAGGTCACGTAG** |
| **MMP14** | **CAAGATTGATGCTGCTCTCTTC** | **ACTTTGATGTTCTTGGGGTACT** |
| **E-cadherin** | **GCCCTGCCAATCCCGATGAAA** | **GGGGTCAGTATCAGCCGCT** |
| **Vimentin** | **GACGCCATCAACACCGAGTT** | **CTTTGTCGTTGGTTAGCTGGT** |
| **ZEB1** | **TTACACCTTTGCATACAGAACCC** | **TTTACGATTACACCCAGACTGC** |
| **SNAIL** | **CACATCCTTCTCACTGCCATGGAATT** | **GCTGCCCTCCCTCCACAGAAAT** |
| **SLUG** | **TGTGACAAGGAATATGTGAGCC** | **TGAGCCCTCAGATTTGACCTG** |
| **RND3** | **TGCGGACAGATGTTAGTACATT** | **AACGTGAAAAATGTCTCTGACG** |
| **FUT3** | **CATCTCAAGGTGGACGTGTAC** | **GAAGGCCAGGTAGAACTTGTAC** |
| **FUT3-exon2** | **GAGGCTGCCATATATCCAGGG** | **AGAGACTCAAAACACAGGCCC** |
| **FUT3-Minigene** | **TCCAACAGAGAAAGCAGGCA** | **GACACACGCAGGTAGGAGAA** |
| **FUT3-Splicing variants** | **CCATGGCCTCCTGGTGAGCTG** | **CCTAGGGGATCCAGTGGCATCG** |
| **IL11** | **GTGGCCAGATACAGCTGTC** | **GAATTTGTCCCTCAGCTGTG** |
| **TCTA** | **AATACAGTGACCGGGTTGTATC** | **ATTCTCTGTGGGTTTTGAGAGG** |
| **PXDN** | **GGATTCTTGACCATCAATGACG** | **GAACATTCACACTGAGCACCAT** |
| **GALNT5** | **TAATGTGGCTTTGGGTAAATGC** | **GGCTATACACCATTCTCCACAT** |
| **TSSK2** | **GACGGATCTACATCATCATGGA** | **CAGACAGCTTGATGTTGAAGTC** |
| **KCTD14** | **ATGTTCTCTAGCTTAGCCAAGG** | **GACCAAAGGCTTGATTTCGTAG** |
| **C10orf95** | **TGCTCACCTGCACCTACCT** | **CGTAGAAGCGGTGGTATTCC** |
| **BHLHB9** | **GTAACTCCAGCTGTGTTAGGAT** | **CCTGATCCTGATTTGACGTCTA** |
| **MAGEA2** | **AGGTGCCTGCTGCCGACTC** | **CTTGGTTGCTGGAGCCCTCATC** |
| **MAGEA6** | **AGGTGCCTGCTGCCGAGTC** | **AGGTCAGGGAAGGTGCTTGGC** |
| **PLGLB1** | **AATACCCAGGGGCCTTCACT** | **TGCCCTGCAGGTGAATTCTT** |
| **SAT1** | **CTGCGGCTGATCAAGGAGCTG** | **GGCAGTGGTAAAAGGGGTGCTC** |
| **SERF1A** | **TCAACGAGAACTTGCCCGC** | **GGCAGTCAAGCTATCCTCTTTCC** |
| **ZNF117** | **TGGCCAGAGCAGAACATAAGAG** | **TGTGCTGTTTACACTCAACCAC** |
| **PAK6** | **GAACTTCCAGCACCGTGTCCAC** | **CCGCAGTGTGTCCAGGATGTTC** |
| **GAPDH** | **GCACCGTCAAGGCTGAGAAC** | **TGGTGAAGACGCCAGTGGA** |
| **18s** | **AGTCCCTGCCCTTTGTACACA** | **CGATCCGAGGGCCTCACTA** |
