# Supplementary material for: The DDX39B/FUT3/TGFβR-I axis promotes tumor metastasis and EMT in colorectal cancer
Source: Cell Death Dis. 2021 Jan 12;12(1):74. doi: 10.1038/s41419-020-03360-6 (PMC7803960; doi:10.1038/s41419-020-03360-6)
Supplement: Supplementary file 13 — Supplementary Table 2 [file 41419_2020_3360_MOESM13_ESM.docx]

**Supplementary Table 2. Sequences of siRNAs.**

| **Name** | **Sense(5’to3’)** | **Antisense(5’to3’)** |
| --- | --- | --- |
| **Control** | **UUCUCCGAACGUGUCACGUTT** | **ACGUGACACGUUCGGAGAATT** |
| **siDDX39B_1** | **CUCGGUAUCAGCAGUUUAATT** | **UUAAACUGCUGAUACCGAGTT** |
| **siDDX39B_2** | **GCCUCAACCUCAAACACAUTT** | **AUGUGUUUGAGGUUGAGGCTT** |
| **shDDX39B** | **GCCUCAACCUCAAACACAUTT** | **AUGUGUUUGAGGUUGAGGCTT** |
| **siFUT3_1** | **GGACAUGGCCUUUCCACAUTT** | **AUGUGGAAAGGCCAUGUCCTT** |
| **siFUT3_2** | **GGAUAUCAUGUCCAACCCUTT** | **AGGGUUGGACAUGAUAUCCTT** |
